# Long non-coding RNA *HOXB-AS3* promotes myeloid cell proliferation and its higher expression is an adverse prognostic marker in patients with acute myeloid leukemia and myelodysplastic syndrome

**DOI:** 10.1186/s12885-019-5822-y

**Published:** 2019-06-24

**Authors:** Huai-Hsuan Huang, Fei-Yun Chen, Wen-Chien Chou, Hsin-An Hou, Bor-Sheng Ko, Chien-Ting Lin, Jih-Luh Tang, Chi-Cheng Li, Ming Yao, Woei Tsay, Szu-Chun Hsu, Shang-Ju Wu, Chien-Yuan Chen, Shang-Yi Huang, Mei-Hsuan Tseng, Hwei-Fang Tien, Ruey-Hwa Chen

**Affiliations:** 1 Division of Hematology, Department of Internal Medicine, National Taiwan University Hospital, Taipei, Taiwan; 2 Department of Laboratory Medicine, National Taiwan University Hospital, National Taiwan University, Taipei, Taiwan; 3 Doctoral Degree Program in Translational Medicine, National Taiwan University and Academia Sinica, Taipei, Taiwan; 4 Institute of Biological Chemistry, Academia Sinica, Taipei, Taiwan; 5 Taicheng Stem Cell Therapy Center, National Taiwan University, Taipei, Taiwan

**Keywords:** *HOXB-AS3*, Acute myeloid leukemia, Myelodysplastic syndrome

## Abstract

**Background:**

Long non-coding RNAs (lncRNAs) represent the majority of cellular transcripts and play pivotal roles in hematopoiesis. However, their clinical relevance in acute myeloid leukemia (AML) and myelodysplastic syndrome (MDS) remains largely unknown. Here, we investigated the functions of *HOXB-AS3*, a lncRNA located at human *HOXB* cluster, in the myeloid cells, and analyzed the prognostic significances in patients with AML and MDS.

**Methods:**

shRNAs were used to downregulate *HOXB-AS3* in the cell lines and the effect was evaluated by quantitative polymerase chain reaction. The proliferation of the cell lines was illustrated by proliferation and BrdU flow assays. Further, we retrospectively analyzed the *HOXB-AS3* expression in 193 patients with AML and 157 with MDS by microarray analysis, and evaluated its clinical importance*.*

**Results:**

Downregulation of *HOXB-AS3* suppressed cell proliferation. Mechanistically, *HOXB-AS3* potentiated the expressions of several key factors in cell cycle progression and DNA replication without affecting the expressions of *HOX* genes. In AML, patients with higher *HOXB-AS3* expression had shorter survival than those with lower *HOXB-AS3* expression (median overall survival (OS), 17.7 months versus not reached, *P* <  0.0001; median relapse-free survival, 12.9 months versus not reached, *P* = 0.0070). In MDS, patients with higher *HOXB-AS3* expression also had adverse prognosis compared with those with lower *HOXB-AS3* expression (median OS, 14.6 months versus 42.4 months, *P* = 0.0018). The prognostic significance of *HOXB-AS3* expression was validated in the TCGA AML cohort and another MDS cohort from our institute. The subgroup analyses in MDS patients showed that higher *HOXB-AS3* expressions could predict poor prognosis only in lower-risk (median OS, 29.2 months versus 77.3 months, *P* = 0.0194), but not higher-risk group.

**Conclusions:**

This study uncovers a promoting role of *HOXB-AS3* in myeloid malignancies and identifies the prognostic value of *HOXB-AS3* expression in AML and MDS patients, particularly in the lower-risk group.

**Electronic supplementary material:**

The online version of this article (10.1186/s12885-019-5822-y) contains supplementary material, which is available to authorized users.

## Background

Acute myeloid leukemia (AML) and myelodysplastic syndrome (MDS) are two myeloid malignancies [1] which share similar somatic gene mutations [2]. A portion of MDS patients eventually progresses to AML as the disease progresses. Several prognostic models have been developed to better risk stratify AML and MDS patients, such as European Leukemia Net (ELN) risk classification for AML [3] and international prognostic scoring system (IPSS) or revised IPSS (IPSS-R) for MDS [4–8]. However, patients may have different prognosis even in the same risk group [9–12]. Exploration of more markers that have prognostic significance are warranted to better risk stratify patients with the diseases.

Long non-coding RNAs (lncRNAs) are transcripts longer than 200 nucleotides without protein coding ability. The functions of most lncRNAs remain poorly characterized, but some of them have been demonstrated to be involved in the hematopoiesis [13–15]. For example, the expression levels of *HOTAIRM1* and *NEAT1* are elevated during myeloid differentiation, and downregulation of *HOTAIRM1* or *NEAT1* delays myeloid maturation [13, 14]. Expression of *XIST*, a lncRNA at X chromosome, inactivates X chromosome in the female cells. The female mice with deletion of *Xist* develop a highly aggressive disease mimicking MDS/MPN [15]. However, the roles of lncRNAs in MDS remain largely unknown [16], and only a few research investigated the role of lncRNAs in de novo AML [17–19]. In the research aimed to find prognostic biomarkers in acute myeloid leukemia (AML), we found expression of *HOXB-AS3*, a lncRNA located at human *HOXB* cluster, is a potential risk factor. However, its clinical relevance and pathogenesis in AML and MDS remain to be determined.

Here, we demonstrate that high expression of *HOXB-AS3* is an adverse prognostic factor for both de novo AML and primary MDS patients. Furthermore, the expression of *HOXB-AS3* promotes cell proliferation in myeloid cells.

## Methods

### Patients

We retrospectively included the adult patients with newly diagnosed primary MDS and de novo AML at the National Taiwan University Hospital (NTUH) from 1992 to 2010. Among them, 157 MDS and 193 AML patients, who had available cryopreserved bone marrow (BM) cells for RNA array analysis and comprehensive clinical information, were recruited for this study. The Cancer Genome Atlas (TCGA) AML cohort available on the TCGA website (https://cancergenome.nih.gov/) and an independent cohort of 30 MDS patients subsequently diagnosed between January 2011 and May 2012 at the NTUH was served as the validation cohorts.

All patients with AML other than acute promyelocytic leukemia (non-APL AML, *n* = 174) underwent standard induction chemotherapy (Idarubicin 12 mg/m^2^ per day for two to 3 days and Cytarabine 100 mg/m^2^ per day for five to 7 days), and consolidation chemotherapy with two-to-four courses of high-dose Cytarabine (2000 mg/m^2^ every twelve hours for 4 days, total eight doses), with or without an anthracycline (idarubicin or mitoxantrone), after they achieved complete remission (CR) as described in our previous studies [20]. Nineteen APL patients received concurrent all-trans retinoic acid (ATRA) and Idarubicin or Mitoxantrone as induction chemotherapy and ATRA with Idarubicin, Mitoxantrone or high dose Cytarabine as consolidation chemotherapy when they achieved CR. If the patients had relapsed or refractory AML, or adverse prognostic factors at diagnosis, such as adverse-risk cytogenetic abnormalities or somatic gene mutations, they underwent allogeneic hematopoietic stem cell transplantation (allo-HSCT) when they had feasible hematopoietic stem cell donors.

In the NTUH MDS training cohort, most MDS patients (70.7%) only received supportive care. Eight patients (5.1%) received AML-directed intensive chemotherapies, 18 (11.5%) received hypomethylating agent (azacitadine or decitabbine), and 20 patients (12.7%) underwent allo-HSCT.

The BM samples from AML and MDS patients were collected at diagnosis, and the mononuclear cells were isolated by Ficoll-Hypaque gradient centrifugation and cryopreserved as previously described [21, 22]. The BM cells from 20 healthy transplantation donors were used as the normal controls to compare the gene expressions with those of AML and MDS patients. This study was approved by the Institutional Review Board of NTUH (IRB number: 201507084RINA and 201503072RINC). All the patients have signed informed consents for the collection of samples and clinical information.

### Analysis of cytogenetic abnormalities and gene mutations

The BM cells were harvested directly or after one to 3 days of un-stimulated cultures. The metaphase chromosomes were banded by the G-banding method as previously described. [23] The determination of mutations in *NPM1* [21, 24], *AML1* (*RUNX1*) [25], *ASXL1* [26], *DNMT3A* [20], *EZH2* [27], *IDH2* [28], *NRAS* [29], *KRAS* [29], *TP53* [30], *SETBP1* [31], *SRSF2* [32], *TET2* [33], *MLL*/PTD [34], *SF3B1* [35], *U2AF35* [36], and *ZRSR2* [35] was performed as described previously.

### Microarray experiments and analysis

The raw data of TCGA AML cohort was downloaded from TCGA website (https://cancergenome.nih.gov/). The detail methods of microarrays for NTUH AML and NTUH MDS cohorts were described in Additional file 1.

The expression levels of two transcript clusters, TC17002254.hg.1 and TC17002858.hg.1, on Affymetrix GeneChip® HTA 2.0 represent *HOXB-AS3* (NCBI Reference Sequence: NC_000017.11) expression. TC17002254.hg.1 detects variants 1 to 5 of *HOXB-AS3*, and TC17002858.hg.1 detects all variants of *HOXB-AS3*. Because TC17002858.hg.1 detects all *HOXB-AS3* variants and the expression pattern was similar between the two transcript clusters, expression of TC17002858.hg.1 was used to stratify patients.

### Cell lines, cell cultures, and associated experiments

OCI/AML3 and TF-1 were human myeloid leukemia cell lines. TF-1 cell line (BCRC number 60323) was purchased from Bioresource Collection and Research Center (BCRC), Hsinchu, Taiwan on Sep 29, 2014. BCRC (http://www.bcrc.firdi.org.tw/) is a nation-wide cell bank in Taiwan, and it provides the service of preservation, identification, and selling of cell lines. OCI/AML3 was a gift from Dr. Minden (Ontario Cancer Institute/Princess Margaret Hospital, Canada) in 2008. The detailed methods of cell cultures, constructions of lentiviral vectors with shRNA and lncRNA, lentiviral production, lentiviral infections, quantitative real time PCR, proliferation assay and nuclear-cytoplasm fractionation were described in Additional file 1.

### BrdU flow assay

BD Pharmingen™ APC BrdU Flow Kits (Cat. NO. 552598) was used for BrdU flow assay. Cells were incubated with 10 μM BrdU at 37 °C for three hours, and then harvested for BrdU flow assay. The detailed method was described in the user manual of the manufacture. The flow cytometry was performed on LSR II (BD Bioscience, San Jose, CA) through the service provided by the Flow Cytometric Analyzing and Sorting Core Facility at the NTUH, and on FACS Canto II (BD Bioscience, San Jose, CA) through the service provided by the Molecular and Immune Function Laboratory at Tai Cheng Stem Cell Therapy Center at the National Taiwan University.

### Statistical analysis

Mann-Whitney test was used to calculate the significance if the continuous data were not normally distributed, and Kruskal-Wallis test was used for comparing the difference between more than two groups. Chi-square test was used to calculate the significance of association between *HOXB-AS3* expression and other categorical parameters, including sex, IPSS risk groups, 2016 WHO subtypes, cytogenetic abnormalities, and gene mutations. Fisher exact test was used if any expected value of the contingency table was less than five.

For patients with de novo AML in the NTUH AML cohort, overall survival (OS) was measured from the date of diagnosis to the date of last follow-up or death. The patients were censored on the date of last follow-up if they were alive. Relapse free survival (RFS) was defined from the date of complete remission to the date of relapse, last follow-up, or death. Relapse and death were defined as events in the RFS analysis. If the patients were alive and in complete remission, they would be censored. For patients with primary MDS, OS was measured from the date of diagnosis to the date of last follow-up or death. The patients were censored on the date of last follow-up if they were alive.

Kaplan-Meier (KM) estimation was used to plot survival curves, and log-rank tests were used to calculate the difference of OS and RFS between different groups in AML patients and OS in MDS patients. Median follow-up duration was calculated by reverse KM estimation. Multivariate Cox proportional hazard regression analysis was used to investigate independent prognostic factors for OS. A *P* value less than 0.05 was considered statistically significant. All statistical analyses were performed with MedCalc® 15.6.1 software (https://www.medcalc.org/).

## Results

### *HOXB-AS3* is an anti-sense lncRNA in the *HOXB* cluster

**Fig. 1:**
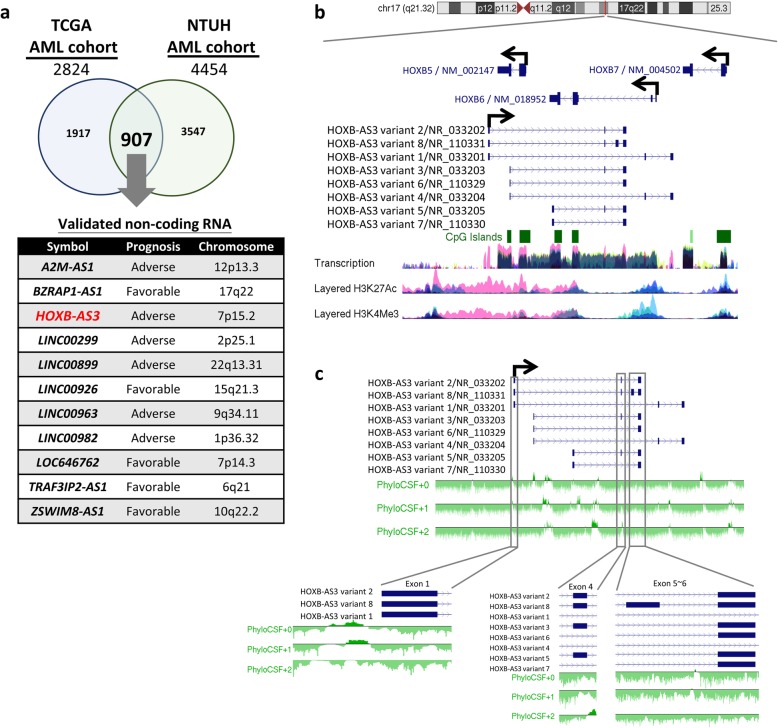
Identification of *HOXB-AS3* as a prognostic biomarker and overview of its locus in human *HOXB*. **a** The numbers of genes influenced overall survival in TCGA AML cohort and NTUH AML cohort. **b** The neighborhood of *HOXB-AS3* locus in human *HOXB* cluster. The genomic data from ENCODE project was taken from UCSC genome browser. The exon localizations of eight *HOXB-AS3* isoforms and *HOXB5*, *HOXB6*, and *HOXB7* genes are shown. Arrows indicate the transcription direction of these genes. The NCBI Reference Sequence (RefSeq) numbers were labeled in the end of each variant. **c** PhyloCSF analysis for predicting the noncoding nature of *HOXB-AS3*. Exon 1, exon 4, exon 5 and exon 6 are zoom-in to see the details of PhyloCSF scores of the specific exons. All PhyloCSF scores are negative in the exons of *HOXB-AS3*

In order to find lncRNAs with significant prognostic implications in myeloid malignancies, we analyzed the microarrays data from two cohorts: one AML cohort from the NTUH (NTUH AML cohort), and another one from The Cancer Genome Atlas (TCGA AML cohort) [37]. The expression of 2824 probes in TCGA AML cohort and 4454 transcript clusters in NTUH AML cohort had prognostic significance (Fig. 1a). The intersect between the two cohorts was 907 probes/transcript clusters, among which eleven lncRNAs were validated as non-coding RNAs according to National Center for Biotechnology Information (NCBI) database (Fig. 1a). Among these lncRNAs, *HOXB-AS3* is particularly interesting, because of the crucial roles of *HOX* genes in cell proliferation, hematopoiesis and leukemogenesis [36, 38, 39] and the clinical significance of an anti-sense lncRNA in the *HOX* clusters, i.e., *HOTAIRM1*, in leukemia [40]. Since the role of lncRNAs in myeloid malignancies remains unclear, the following studies were focused on the role of *HOXB-AS3* in myeloid malignancies.

*HOXB-AS3* is a lncRNA located at human *HOXB* cluster on the chromosome 17q21.32 and has eight transcriptional variants generated by alternative splicing (Fig. 1b). The entire gene is overlapped with *HOXB5* and *HOXB6* genes, and the transcriptional direction is antisense to the overlapping *HOXB* genes (Fig. 1b). The exons of *HOXB-AS3*, except for exon 2, are not overlapped with the exons of *HOXB5* or *HOXB6* (Fig. 1b). By using PhyloCSF pipeline, *HOXB-AS3* is predicted to be a noncoding RNA (Fig. 1c), and the coding probability is low (0.126) by Coding Potential Assessment Tool (CPAT) [41]. Therefore, *HOXB-AS3* is an anti-sense lncRNA in the *HOXB* cluster, similar to *HOTAIR* in the *HOXC* cluster, and *HOTTIP* and *HOTAIRM1* in the *HOXA* cluster [13, 42, 43].

A similar anti-sense transcript, *Hoxb5os* (NCBI Reference Sequence: NR_131758.1), exists in the mouse *HOXB* cluster on chromosome 11 (NCBI Reference Sequence: NC_000077.6 Chromosome 11 Reference GRCm38.p4 C57BL/6 J), and is also predicted to be a lncRNA (Additional file 1: Figure S1). We aligned the sequences of human *HOXB-AS3* with that of mouse *Hoxb5os* by plalign (http://fasta.bioch.virginia.edu/) and observed two conserved regions (Additional file 1: Figure S2), one in exon 1 and the other in exon 6 of *HOXB-AS3*. These analyses indicated that *HOXB-AS3* might be an evolutionarily conserved anti-sense transcript in the *HOXB* cluster in mammals.

### *HOXB-AS3* is a cytoplasmic lncRNA and promotes cell proliferation by increasing the expressions of genes involved in cell cycle progression and DNA replication

**Fig. 2:**
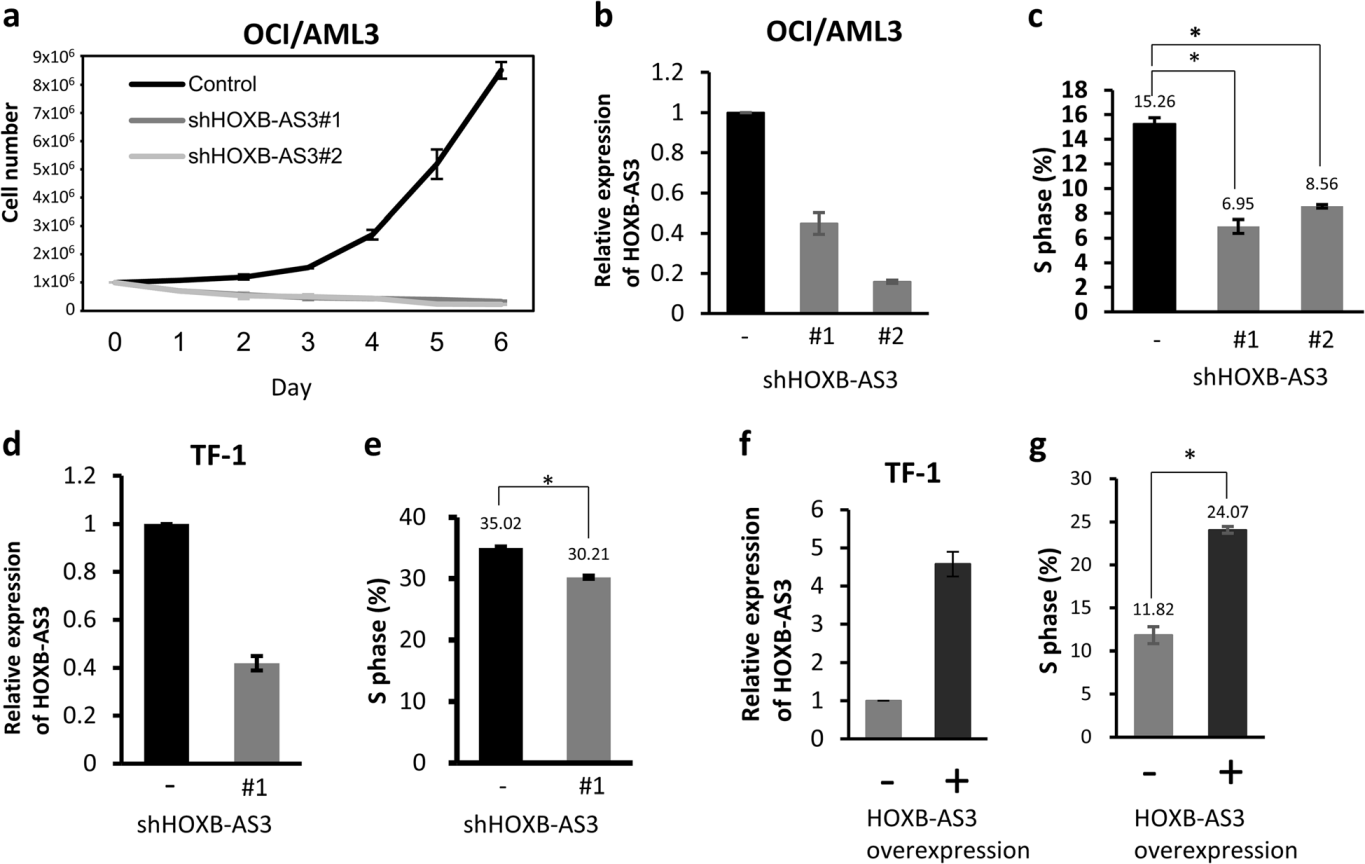
*HOXB-AS3* promotes cell proliferation in myeloid cells**. a** Proliferation assay of OCI/AML3 cells infected with lentivirus carrying pLKO-vector (control), shHOXB-AS3#1, or shHOXB-AS3#2. The infected cells were sorted by the expression of GFP. The sorted GFP-positive cells were seeded with a number of 1 × 10^6^ in 5 mL culture medium on Day 0. Cell number was counted for 6 days. *P* values were calculated by Kruskal-Wallis test. **b** Quantitative PCR analysis of *HOXB-AS3* relative to *β2 microglobulin* expression in OCI/AML3 cells stably carrying pLKO-vector (control), shHOXB-AS3#1, or shHOXB-AS3#2 by lentivirus infection. **c** The percentage of OCI/AML3 cells in the S phase of cell cycle. The result was derived from triple repeats. *P* values were calculated by Kruskal-Wallis test. **d** Quantitative PCR analysis of *HOXB-AS3* relative to *β2 microglobulin* expression in TF-1 cells stably carrying pLKO-vector (control) or shHOXB-AS3#1 by lentivirus infection. **e** The percentage of TF-1 cells in the S phase of cell cycle. The result was derived from triple repeats. *P* values were calculated by Student t test. **f** Quantitative PCR analysis of *HOXB-AS3* relative to *β2 microglobulin* expression in TF-1 cells infected with lentivirus carrying pLAS5w.Pbsd vector (control) or pLAS5w.Pbsd-HOXB-AS3 (*HOXB-AS3* overexpression). **g** The percentage of cells in the S phase of cell cycle. The result was derived from the triple repeats. *P* values were calculated by Student t test. (* meant that *P* value was less than 0.05)

To investigate the function of *HOXB-AS3*, we infected the myeloid cell line OCI/AML3 using lentivirus carrying control or each of the two *HOXB-AS3* shRNAs together with GFP. The infected cells were sorted for proliferation assay. We found that the cells carrying *HOXB-AS3* shRNA grew more slowly than those carrying control shRNA (Fig. 2a). The result suggested the anti-proliferative effect resulted from *HOXB-AS3* depletion. To corroborate this effect, we stably knocked down *HOXB-AS3* in two myeloid cell lines, OCI/AML3 and TF-1 (Fig. 2b and d). Downregulation of *HOXB-AS3* suppressed the proliferation of OCI/AML3 and TF-1 cells by decreasing the cells entering the S phase (Fig. 2c and e**;** Additional file 1: Figure S3a and S3b). Compared with OCI/AML3 cells, the milder effect of *HOXB-AS3* depletion in TF-1 cells was possibly resulted from the very low expression of *HOXB-AS3* in TF-1 cells (Additional file 1: Figure S3d). In the reciprocal experiments, overexpression of *HOXB-AS3* in TF-1 cell line enhanced cell proliferation by promoting cells entering the S phase (Fig. 2f and g**;** Additional file 1: Figure S3c). These findings indicated that *HOXB-AS3* promoted cell proliferation in the myeloid cell lines.

To explore the mechanisms of *HOXB-AS3* in proliferation, we investigated the expression of genes influenced by *HOXB-AS3* depletion through the microarray analysis. We compared the gene expression profiles of OCI/AML3 cells infected by virus carrying shHOXB-AS3 (two stable cell lines infected with different shHOXB-AS3, shHOXB-AS3#1 or shHOXB-AS3#2) with those infected by control virus (two stable cell lines infected with shLacZ or pLKO vector).

**Fig. 3:**
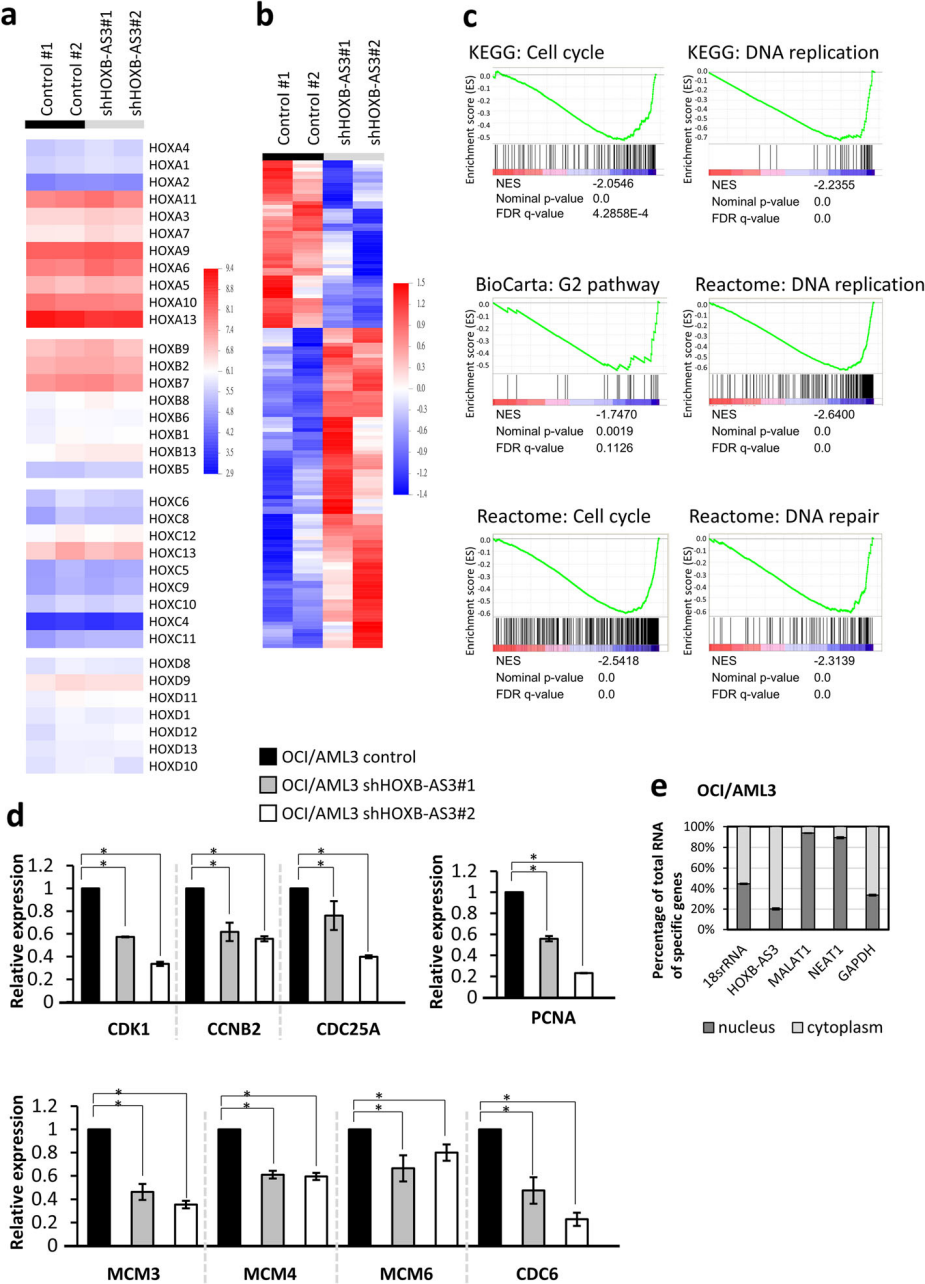
*HOXB-AS3* regulates genes involving in cell cycle progression and DNA replication instead of *HOX* clusters**. a** The expressions of *HOX* genes were not influenced by *HOXB-AS3* expression. RNA was purified from OCI/AML3 infected by lentivirus carrying pLKO-shLacZ (control#1), pLKO-vector (control#2), pLKO-shHOXB-AS3#1 or pLKO-shHOXB-AS3#2. **b** Differentially expressed genes between the control cells and *HOXB-AS3* knockdown cells were revealed by microarray analysis. **c** GSEA analysis of the differentially expressed genes in *HOXB-AS3* knockdown cells compared to the control cells. **d** Quantitative PCR analysis of the expressions of indicated genes in OCI/AML3 cells infected with lentivirus carrying pLKO-vector (control), shHOXB-AS3#1, or shHOXB-AS3#2. Data were derived from the triple repeats of experiments. *P* values were calculated by Kruskal-Wallis test. **e** Quantitative real time PCR analysis of *RNA18S5 RNA* (*18 s rRNA*), *HOXB-AS3*, *MALAT1*, *NEAT1* and *GAPDH* in the nuclear and cytoplasmic fractions of OCI/AML3 cells. (* meant that *P* value was less than 0.05)

We compared the gene expressions of *HOX* clusters between OCI/AML3 cells with and without *HOXB-AS3* depletion because previous studies showed that most lncRNAs in *HOX* clusters are located in the nucleus and regulate the expressions of *HOX* genes in *cis*- or *trans*-manner [42, 43]. However, we found that the expressions of *HOX* clusters were not influenced by *HOXB-AS3* depletion (Fig. 3a). Instead, we identified 147 genes and 790 transcript clusters differentially expressed when *HOXB-AS3* was knocked down in OCI/AML3 cells (Fig. 3b; the criteria were that the fold change of average log2-transformed gene expressions was more than 1.5, and that ANOVA *P* value was less than 0.05. The analysis was computed by BRB array tool.). GSEA analysis revealed that these differentially expressed genes were involved in several pathways associated with cell cycle progression and DNA replication (Fig. 3c and Additional file 1: Figure S4). Wikipathway analysis computed by Affymetrix® TAC also showed that many differentially expressed genes were involved in cell cycle pathway, DNA replication, G1-S transition, and RB pathway (Additional file 1: Figure S5-S8). Moreover, the expressions of these genes were mostly downregulated when *HOXB-AS3* was knocked down, consistent with a promoting function of this lncRNA in cell proliferation.

We validated a set of *HOXB-AS3* regulated genes by RT-qPCR and confirmed that several genes involved in the cell cycle progression (CDK1, CCNB2, and CDC25A), DNA replication (PCNA), and assembly of pre-replicative complex (CDC6, MCM4, MCM6) were indeed downregulated when *HOXB-AS3* was knocked down (Fig. 3d). Conversely, these genes were upregulated when *HOXB-AS3* was overexpressed (Additional file 1: Figure S9). These findings suggested that *HOXB-AS3* induced the expression of a number of genes involving in cell cycle progression and DNA replication to contribute to myeloid cell proliferation.

Furthermore, to identify the subcellular location of lncRNA *HOXB-AS3*, we used nuclear-cytoplasm fractionation to purify RNA from the nucleus and cytoplasm of OCI/AML3 cells, followed by reverse transcription and quantitative real time PCR. Our analysis demonstrated that the majority of *HOXB-AS3* was located at the cytoplasm, different from the nuclear-residing *MALAT1* and *NEAT1* (Fig. 3e) [44, 45]. Therefore, our results suggest that *HOXB-AS3* regulate the expressions of downstream genes through an indirect mechanism, which was different from other anti-sense lncRNAs in the *HOX* clusters.

### Higher *HOXB-AS3* expression is associated with shorter overall survival and progression free survival in AML patients

To determine the clinical significance of *HOXB-AS3* in de novo AML patients, we analyzed the microarrays and clinical data of the NTUH AML cohort and validated the results with TCGA AML cohort. In the NTUH AML cohort, *HOXB-AS3* expression was determined based on the expression levels of TC17002858.hg.1, the transcript cluster representing all variants of *HOXB-AS3* on Affymetrix GeneChip® HTA 2.0. The AML patients were stratified into higher- and lower-expression groups with the median expression of *HOXB-AS3* as the cutoff level. The *HOXB-AS3* levels in the higher-expression group were much higher than those of the healthy donors (Additional file 1: Figure S10; *P* <  0.000001), while the expression levels were similar between lower group and the healthy donors (Additional file 1: Figure S10). The clinical characteristics of the NTUH AML patients were listed in Additional file 1: Table S1 Patients with higher *HOXB-AS3* expression were older and had higher frequencies of mutated *NPM1*/wild *FLT3*-ITD, *MLL*-PTD, and *RUNX1* mutations, but lower frequency of *CEBPAɑ* double mutations than patients with lower expression.

**Fig. 4:**
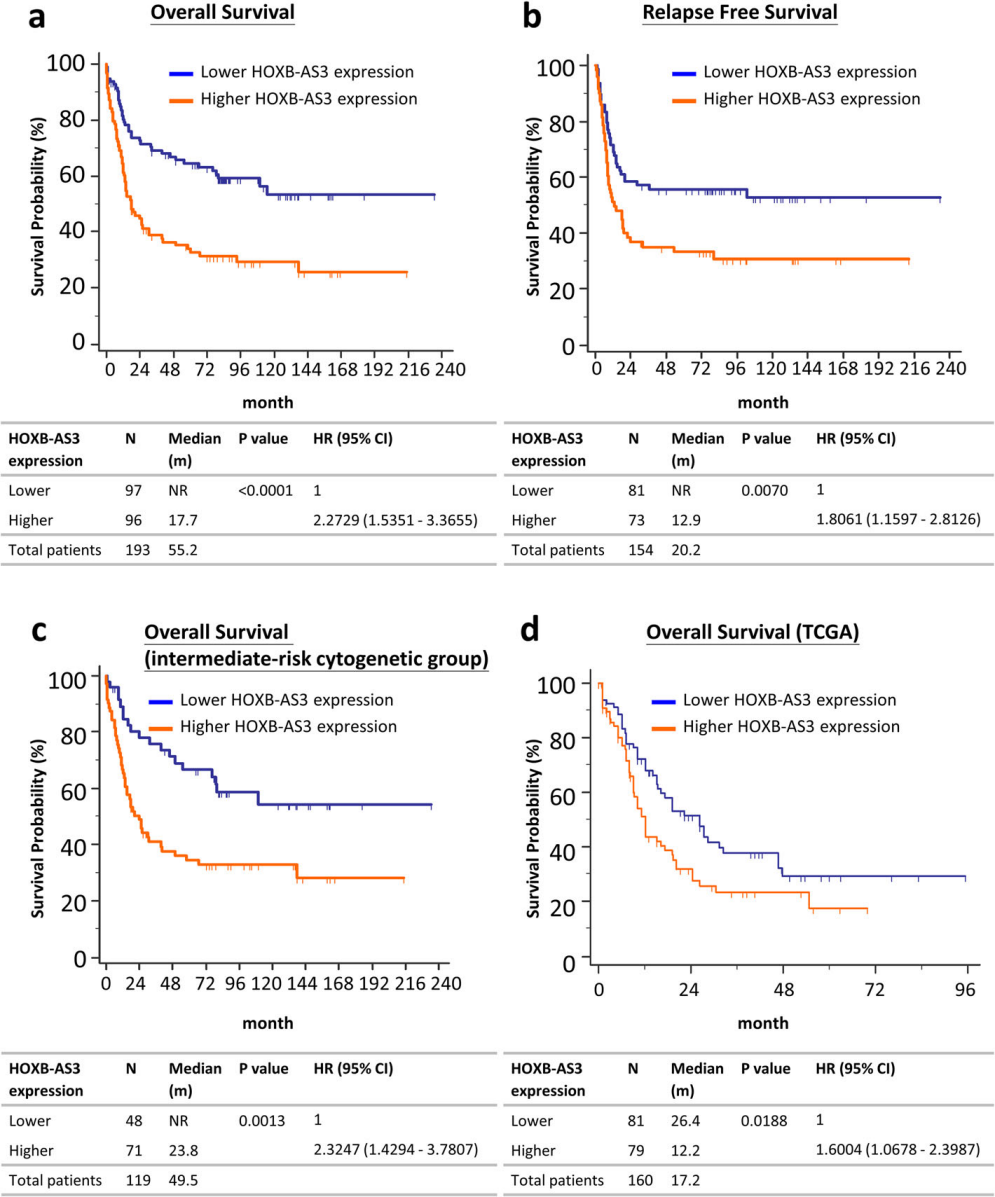
Survival analysis of de novo AML patients stratified by the expressions of *HOXB-AS3***. a** Overall survival (OS) in the NTUH AML cohort. **b** Relapse free survival in the NTUH AML cohort. **c** OS in the AML patients with intermediate-risk cytogenetic changes. **d** OS in the TCGA AML cohort. The patients in the NTUH AML cohort were stratified by the expressions of transcript cluster: TC17002858.hg.1 on Affymetrix GeneChip® HTA 2.0 arrays

Patients with higher *HOXB-AS3* expression had similar complete remission rate to those with lower expression, but had higher relapse rate (Additional file 1: Table S1). With a median follow-up time of 88.1 months, the AML patients with higher *HOXB-AS3* expressions had shorter OS and RFS than those with lower *HOXB-AS3* expressions (Fig. 4a and b; median OS, 17.7 months versus not reached, *P* value < 0.0001; median RFS, 12.9 months versus not reached, *P* value 0.0070, respectively). When we stratified patients according to the expression levels of the other transcript cluster representing *HOXB-AS3*, TC17002254.hg.1, the results were similar (Additional file 1: Figure S11). Subgroup analysis showed that higher *HOXB*-AS3 expression was also a poor-risk factor for OS in the intermediate-risk cytogenetic group (Fig. 4c). We validated our findings in the TCGA AML cohort in whom higher *HOXB-AS3* expression was also an adverse prognostic biomarker (Fig. 4d) [37]. Multivariate analysis showed that higher *HOXB-AS3* expression tended to be an independent adverse prognostic factor for OS in AML patients (*P* = 0.0634, Table 1).

**Table 1:** Univariate and multivariate analyses (Cox regression) for overall survival in AML patients

Variables	Univariate analysis			Multivariate analysis		
	HR	95% CI	*P* value	HR	95% CI	*P* value
Age > 60 years	2.7201	1.5917 to 4.6484	< 0.0001*	2.0425	1.2585 to 3.3148	0.0038*
WBC > 100 k μ/L	1.0529	0.6026 to 1.8399	0.8534	–	–	–
Favorable cytogenetics	0.4552	0.2905 to 0.7133	0.0051*	0.4579	0.2315 to 0.9058	0.0248*
*NPM1*+/ *FLT3*-ITD-	0.5674	0.3346 to 0.9625	0.0839	0.2972	0.1421 to 0.6216	0.0013*
*FLT3*-TKD	1.1062	0.5419 to 2.2580	0.7722	–	–	–
*CEBPα* double mutation	0.3093	0.1839 to 0.5201	0.0030*	0.3893	0.1585 to 0.9561	0.0396*
*PTPN11* mutation	1.2426	0.5008 to 3.0836	0.6042	–	–	–
*KRAS* mutation	1.6638	0.6351 to 4.3587	0.1885	–	–	–
*MLL*/PTD	3.5868	1.0660 to 12.0688	0.0001*	2.3662	1.0555 to 5.3047	0.0365*
*KIT* mutation	1.0609	0.4827 to 2.3319	0.8797	–	–	–
*RUNX1* mutation	2.2459	1.1285 to 4.4697	0.0013*	1.2736	0.6998 to 2.3179	0.4285
*WT1* mutation	1.4062	0.7398 to 2.6728	0.2326	–	–	–
*ASXL1* mutation	0.8468	0.3932 to 1.8236	0.6921	–	–	–
*IDH1* mutation	0.5760	0.2364 to 1.4033	0.3395	–	–	–
*IDH2* mutation	0.9412	0.5109 to 1.7341	0.8492	–	–	–
*TET2* mutation	1.1970	0.6549 to 2.1879	0.5309	–	–	–
*TP53* mutation	4.3752	0.5761 to 33.2257	0.0015*	3.1834	1.0992 to 9.2198	0.0328*
*DNMT3A* mutation	1.3679	0.8133 to 2.3004	0.1908	–	–	–
*HOXB-AS3* expression	2.3003	1.5530 to 3.4072	< 0.0001*	1.6401	0.9727 to 2.7654	0.0634

Age, elder than 60 years versus younger; WBC, higher than 100 k μ/L versus lower; *HOXB-AS3* expression, higher versus lower; *NPM1*+/ *FLT3*-ITD-, mutated *NPM1* without *FLT3*-ITD versus others; other mutations, mutation versus wild type; favorable cytogenetics, favorable versus others

Abbreviations: *HR* hazard ratio, *CI* confidence interval, *MLL/PTD* partial tandem duplication of *MLL* gene

* *P* value < 0.05

We further analyzed the patients with AML other than acute promyelocytic leukemia (non-APL), and stratified them according to the median expression of *HOXB-AS3* (Additional file 1: Figure S12). Patients with higher *HOXB-AS3* expression still had shorter OS than those with lower *HOXB-AS3* expression (Additional file 1: Figure S13a). The subgroup analysis in the intermediate-risk patients based on the 2017 European LeukemiaNet (ELN) risk classification also illustrated adverse prognosis for the patients with higher *HOXB-AS3* expression (Additional file 1: Figure S13b).

### Higher *HOXB-AS3* expression also predicts adverse prognosis in MDS patients

To investigate the clinical relevance of *HOXB-AS3* expressions in primary MDS, we analyzed the microarrays and clinical data of the NTUH MDS training cohort and validation cohort. *HOXB-AS3* expression was determined based on the expression levels of TC17002858.hg.1. According to the expression levels of *HOXB-AS3*, the MDS patients was stratified into four groups, lowest, intermediate low, intermediate high, and highest *HOXB-AS3* expression groups (Additional file 1: Figure S14). Only the patients in the highest group had distinct higher *HOXB-AS3* expressions compared with the healthy donors (Additional file 1: Figure S14; *P* <  0.000001), while patients in other groups had similar *HOXB-AS3* expressions to normal controls.

**Fig. 5:**
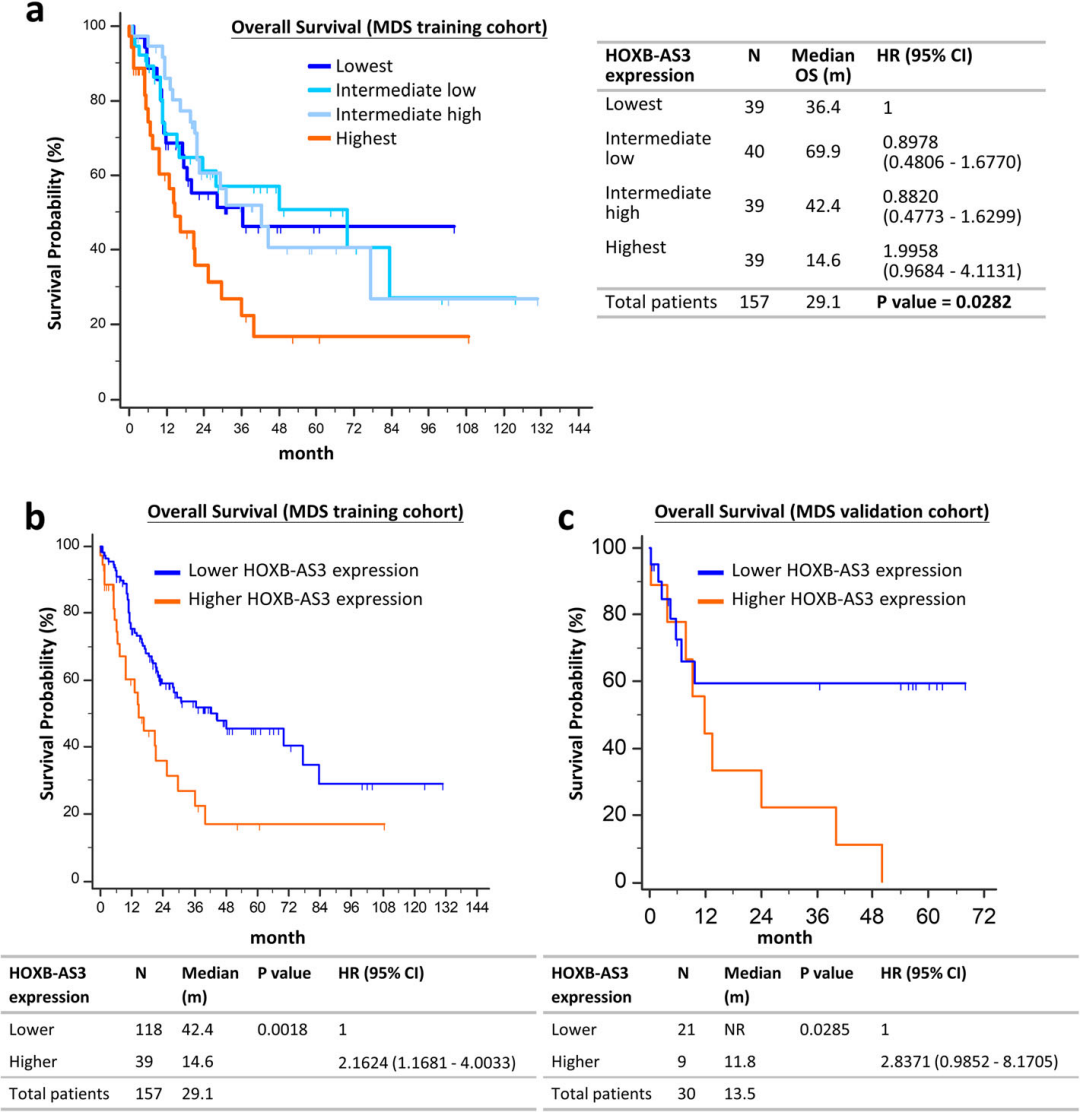
Overall survival of MDS patients stratified by the expressions of *HOXB-AS3***. a** OS in the NTUH MDS training cohort stratified into 4 groups: *HOXB-AS3* expression highest, intermediate high, intermediate low and lowest groups. **b** OS in the NTUH MDS training cohort stratified into 2 groups: *HOXB-AS3* expression higher group (*HOXB-AS3* expression highest group in Fig. 5a) and lower group (*HOXB-AS3* expression lowest, intermediate low, and intermediate high groups in Fig. 5a). **c** OS in the NTUH MDS validation cohort stratified into 2 groups as Fig. 5b. The patients were stratified by the expressions of transcript cluster: TC17002858.hg.1 on Affymetrix GeneChip® HTA 2.0 arrays

With a median follow-up time of 39.2 months, the MDS patients with highest *HOXB-AS3* expressions had the shortest OS compared to others (Fig. 5a). The OS was similar among the patients with lowest, intermediate low, and intermediate high expressions of *HOXB-AS3*. Similar results were obtained when we stratified the patients according to the expressions of another transcript cluster, TC17002254.hg.1, which also represented *HOXB-AS3* (Additional file 1: Figure S15a). If we used the cutoff point between the highest and intermediate high groups (Fig. 5a**)** to stratify the NTUH MDS training cohort into two groups, higher *HOXB-AS3* expression predicted shorter OS in the MDS patients (Fig. 5b). The clinical characteristics of the MDS patients were listed in Additional file 1: Table S2, and the correlations of *HOXB-AS3* expressions and somatic gene mutations were listed in Additional file 1: Table S3. Patients with higher *HOXB-AS3* expression had a high rate of leukemic transformation, higher bone marrow blast percentage and more frequently WHO higher-risk subtypes, but similar distribution of IPSS subtypes, compared to those with lower *HOXB-AS3* expression. Higher *HOXB-AS3* expression was associated with *RUNX1*, *ASXL1*, and *IDH2* mutations. When we applied the same cutoff point to the MDS validation cohort, the prognostic significance of *HOXB-AS3* expression for OS in MDS patients was also confirmed (Fig. 5c and Additional file 1: Figure S15b). Multivariate analysis demonstrated that higher *HOXB-AS3* expression was an independent poor prognostic factor for OS in primary MDS patients, irrespective of other poor prognostic factors, including higher IPSS scores and adverse risk mutations (Table 2).

**Table 2:** Univariate and multivariate analyses (Cox regression) for overall survival in MDS patients

Variables	Univariate analysis			Multivariate analysis		
	HR	95% CI	*P* value	HR	95% CI	*P* value
Age > 60 years	1.7988	1.1298 to 2.8641	0.0223*	1.5433	0.8392 to 2.8383	0.1628
IPSS	3.4616	1.9730 to 6.0735	< 0.0001*	2.5755	1.4292 to 4.6412	0.0016*
*EZH2* mutation	2.0164	0.7036 to 5.7785	0.0709	1.1877	0.4371 to 3.2271	0.7358
*RUNX1* mutation	1.6949	0.7996 to 3.5925	0.0880	0.7265	0.3155 to 1.6731	0.4528
*ASXL1* mutation	1.9909	1.0927 to 3.6274	0.0059*	1.3616	0.6273 to 2.9554	0.4350
*IDH2* mutation	1.5777	0.2748 to 9.0568	0.5195	–	–	–
*ZRSR2* mutation	2.5929	1.0588 to 6.3500	0.0016*	1.9474	0.8973 to 4.2263	0.0918
*U2AF35* mutation	1.0584	0.4771 to 2.3480	0.8861	–	–	–
*TET2* mutation	1.3481	0.6591 to 2.7572	0.3579	–	–	–
*SRSF2* mutation	1.9654	0.9379 to 4.1184	0.0196*	1.5304	0.7107 to 3.2954	0.2769
*SF3B1* mutation	0.7602	0.4237 to 1.3640	0.3970	–	–	–
*TP53* mutation	7.1283	1.2057 to 42.1423	< 0.001*	6.1091	2.3732 to 15.7260	0.0002*
*DNMT3A* mutation	1.2781	0.6513 to 2.5080	0.4344	–	–	–
*HOXB-AS3* expression	2.1624	1.1681 to 4.0033	0.0018*	1.8992	1.0606 to 3.4009	0.0309*

Age, elder than 60 years versus younger; *HOXB-AS3* expression, highest versus others; mutations, mutation versus wild type; IPSS, intermediate-2 and high, versus low and intermediate-1

Abbreviations: *HR* hazard ratio, *CI* confidence interval

* *P* value < 0.05

**Fig. 6:**
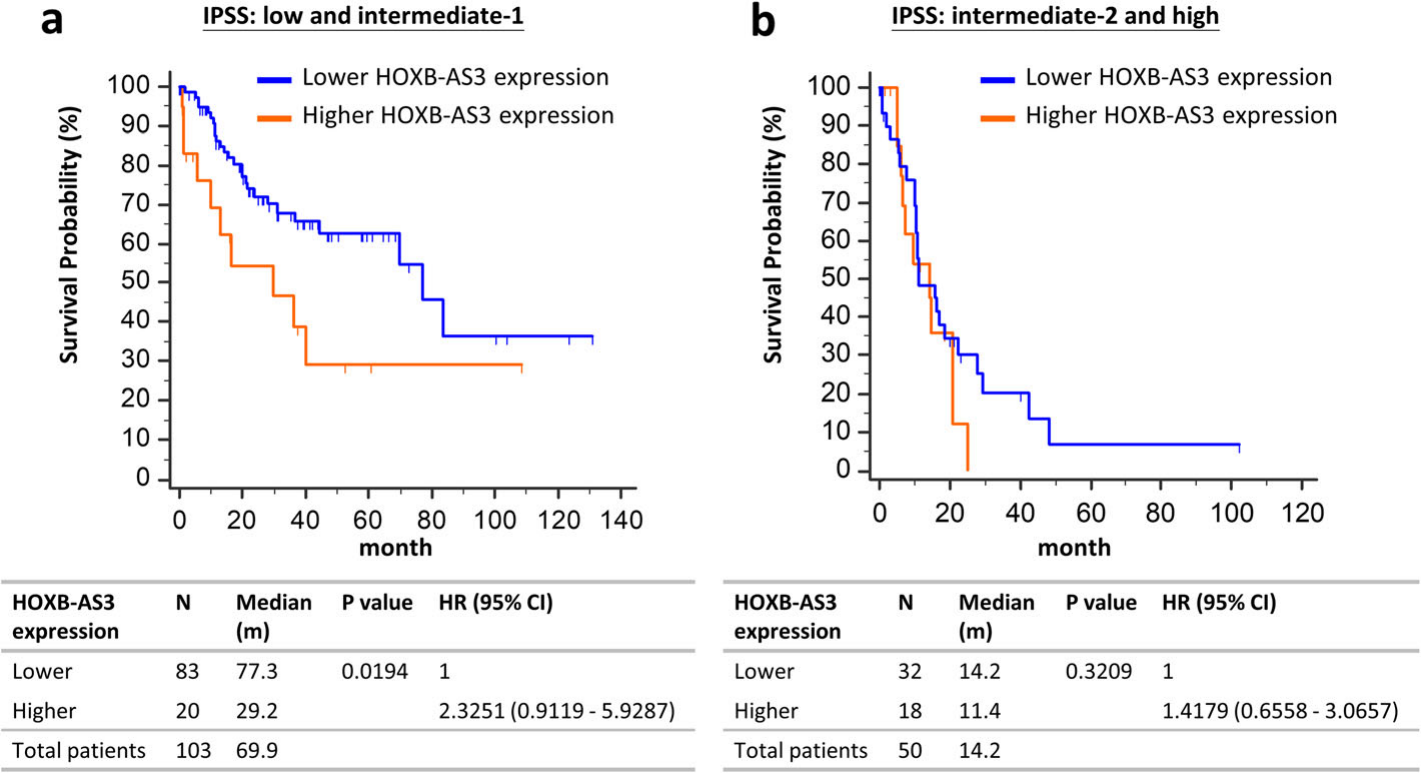
OS of MDS patients stratified by the expressions of *HOXB-AS3* in different IPSS risk groups. **a** OS in the patients with IPSS low or intermediate-1 risk, stratified by *HOXB-AS3* expressions. **b** OS in the patients with IPSS intermediate-2 or high risk, stratified by *HOXB-AS3* expressions. The patients were stratified by the expressions of transcript cluster: TC17002858.hg.1 on Affymetrix GeneChip® HTA 2.0 arrays into 2 groups as Fig. 5b

Furthermore, we analyzed prognostic implications of *HOXB-AS3* expressions in subgroups of patients with different IPSS risk. Among the patients with IPSS lower risk MDS (low and intermediate-1 risks), those with higher *HOXB-AS3* expression had shorter OS than those with lower expression (median, 29.2 months vs 77.3 months, *P* value 0.0194; Fig. 6a). On the other hand, *HOXB-AS3* expression did not influence OS in the patients with IPSS higher risk MDS (intermediate-2 and high risks; Fig. 6b). Therefore, high *HOXB-AS3* expression could further identify a subgroup of patients with high risk in the IPSS lower risk group.

## Discussion

In this study, we reported the clinical relevance of lncRNA *HOXB-AS3* in de novo AML and primary MDS. We demonstrated that higher *HOXB-AS3* expression was an adverse prognostic factor for AML and MDS patients. To our knowledge, *HOXB-AS3* represents the first reported lncRNA whose expression is able to predict the prognosis of MDS patients. We also found that *HOXB-AS3* expression could further stratify IPSS lower risk patients into two subgroups with distinct prognosis. This would help us identify the IPSS lower risk patients who need to be treated aggressively.

In addition to identifying the prognostic value of *HOXB-AS3* expression in myeloid malignancies, we explored the biological function of *HOXB-AS3*. We revealed that *HOXB-AS3* promoted the proliferation of myeloid cells. Previous studies showed that anti-sense lncRNAs in *HOX* clusters influence the expressions of *HOX* genes by *trans*- or *cis*-regulation [13, 42, 43]. For examples, *HOTAIR trans*-regulates the expression of *HOXD* genes through PRC2 complex [42], whereas *HOTAIRM1 cis*-regulates the expressions of *HOXA1* and *HOXA4* genes [13]. Given that expressions of *HOX* genes are important in cell proliferation, hematopoiesis and leukemogenesis [36, 38, 39], it is possible that *HOXB-AS3* promotes cell proliferation by regulating the expressions of certain *HOX* genes. However, our microarray analysis indicated that downregulation of *HOXB-AS3* in OCI/AML3 cells did not significantly alter the expression of any *HOX* gene (Fig. 3a). Instead, *HOXB-AS3* potentiated the expressions of a set of genes critical for cell cycle progression and DNA replication (Fig. 3c and Additional file 1: Figure S4), and this finding was consistent with its ability to increase the S-phase cell population (Fig. 2c, e, and g). A recent study showed that knocking down *HOXB-AS3* reduces the cells in S phase and the ability in colony formation, which is consistent with our findings [46]. Further, they demonstrated that *HOXB-AS3* binds EBP1 to increase the EBP1-NPM1 complex [46]. Accordingly, overexpression of *HOXB-AS3* increases the transcription of rRNA and de novo protein synthesis [46]. This mechanism might explain the effect of *HOXB-AS3* on cell proliferation.

The clinical relevance of *HOXB-AS3* in hematopoietic diseases remains poorly characterized. A previous study reported a positive correlation of *HOXB-AS3* expression with *NPM1* mutations in AML patients [47]. In our study, we not only illustrated high *HOXB-AS3* expression as a poor prognostic biomarker in AML and MDS, but also disclosed its promotion effect on cell proliferation in two myeloid cell lines (Fig. 2). Further, we showed that higher *HOXB-AS3* expression was an adverse prognostic marker in IPSS lower risk patients, but not higher risk ones. It may indicate that higher expressions of *HOXB-AS3* influences the prognosis through enhancing proliferation of the abnormal hematopoietic cells in IPSS lower-risk patients, but has no implication when the patients already have many risk factors as in IPSS higher risk patients. These findings imply that *HOXB-AS3* may have distinct clinical relevance in different myeloid malignancies.

Of note, a recent study reported that *HOXB-AS3* can encode a small peptide to influence the alternative splicing of pyruvate kinase M, thereby inhibiting the proliferation of colon cancer cell lines [48]. In addition, *HOXB-AS3* expression is downregulated in colorectal cancer (CRC) tissues and is correlated with favorable prognosis for CRC patients [48]. The seemingly discrepancies between this previous study and our findings are likely due to the different variants of *HOXB-AS3* used. The previous study investigated exclusively *HOXB-AS3* variant 1 (NR_033201.2), and the small peptide is encoded from the last two exons of this variant [48]. However, in leukemia cell lines, variant 1 is expressed at a very low level, and the majority of *HOXB-AS3* transcripts are variants 2/3/5 (Additional file 1: Figure S16), which do not contain the last two exons (Fig. 1b) and therefore cannot encode the small peptide. Because of the abundant expression of variants 2/3/5, we used the longest variant, variant 2, for the overexpression studies and results are consistent with the conclusions derived from the knockdown studies, in which the two *HOXB-AS3* shRNAs target variants 2, 3, 5, 8 and variants 2, 3, 5, 6, 7, 8, respectively. Therefore, the current and previous studies suggest the existence of variant-specific functions of *HOXB-AS3*. The relative abundance of *HOXB-AS3* variants could determine its context-dependent roles in different cancer types.

## Conclusions

Our study identifies that higher expression of *HOXB-AS3* is an adverse prognostic marker for both de novo AML and primary MDS patients. Functionally, *HOXB-AS3* promotes the proliferation of myeloid cells through upregulating the expressions of a set of genes critical for cell cycle progression and DNA replication. *HOXB-AS3* can be a potential target for novel therapy in MDS and AML patients with higher *HOXB-AS3* expression.

## Additional file

Additional file 1: **Table S1.** Correlation of HOXB-AS3 expression with clinical characteristics and frequent somatic gene mutations in de novo AML patients. **Table S2.** Correlation of HOXB-AS3 expression with clinical characteristics in MDS patients. **Table S3.** Correlation of HOXB-AS3 expression with frequent gene mutations in MDS patients. **Figure S1.** Overview of Hoxb5os in mouse HOXB cluster. **Figure S2.** Alignment of mouse Hoxb5os and human HOXB-AS3. **Figure S3.** HOXB-AS3 promotes S phase entering in the cell cycle regulation. **Figure S4.** GSEA pathway analysis of the differentially expressed genes in HOXB-AS3 knockdown cells compared to the control cells. **Figure S5.** Cell cycle pathway from Wikipathway analysis of downstream pathways affected by downregulation of HOXB-AS3 in the myeloid cell lines. **Figure S6.** DNA replication pathway from Wikipathway analysis of downstream pathways affected by downregulation of HOXB-AS3 in the myeloid cell lines. **Figure S7.** RB pathway from Wikipathway analysis of downstream pathways affected by downregulation of HOXB-AS3 in the myeloid cell lines. **Figure S8.** G1-S pathway from Wikipathway analysis of downstream pathways affected by downregulation of HOXB-AS3 in the myeloid cell lines. **Figure S9.** Quantitative PCR analysis of the expressions of indicated genes in TF-1 cells infected with lentivirus carrying pAS5.1w-Pbsd (control), or pAS5.1w-Pbsd-HOXB-AS3 (HOXB-AS3 overexpression). **Figure S10.** HOXB-AS3 expressions of AML patients and health donors. **Figure S11.** Survival analysis of AML patients stratified by the expressions of HOXB-AS3. **Figure S12.** HOXB-AS3 expression of non-APL AML patients and health donors. **Figure S13.** Survival analysis of non-APL AML patients stratified by the expressions of HOXB-AS3 in the NTUH AML cohort. **Figure S14.** HOXB-AS3 expressions of MDS patients and health donors. **Figure S15.** Overall survival of MDS patients stratified by the expressions of HOXB-AS3. Figure S16. Quantitative PCR analysis of the expressions of different variants in TF-1 and OCI/AML3 cell lines. (PDF 2900 kb)

## Data Availability

The raw data of TCGA AML cohort was downloaded from TCGA website (https://cancergenome.nih.gov/). The datasets supporting the conclusions of this article are available in NCBI’s Gene Expression Omnibus (GEO), and were accessible through GEO Series accession number GSE114823 (https://www.ncbi.nlm.nih.gov/geo/query/acc.cgi?acc=GSE114823), GSE114868 (https://www.ncbi.nlm.nih.gov/geo/query/acc.cgi?acc=GSE114868), and GSE114869 (https://www.ncbi.nlm.nih.gov/geo/query/acc.cgi?acc=GSE114869).

## Abbreviations

allo-HSCT: allogeneic hematopoietic stem cell transplantation
AML: Acute myeloid leukemia
APL: Acute promyelocytic leukemia
ATRA: All-trans retinoic acid
BM: Bone marrow
CPAT: Coding Potential Assessment Tool
CR: Complete remission
CRC: Colorectal cancer
ELN: European Leukemia Net
GEO: Gene Expression Omnibus
IPSS: International prognostic scoring system
IPSS-R: Revised international prognostic scoring system
KM: Kaplan-Meier
lncRNAs: long non-coding RNAs
MDS: Myelodysplastic syndrome
MPN: Myeloproliferative neoplasms
NCBI: National Center for Biotechnology Information
NTUH: National Taiwan University Hospital
OS: Overall survival
RFS: Relapse free survival
TCGA: The Cancer Genome Atlas
