# Supplementary material for: Long non-coding RNA HOXB-AS3 promotes myeloid cell proliferation and its higher expression is an adverse prognostic marker in patients with acute myeloid leukemia and myelodysplastic syndrome
Source: BMC Cancer. 2019 Jun 24;19:617. doi: 10.1186/s12885-019-5822-y (PMC6591843; doi:10.1186/s12885-019-5822-y)
Supplement: Supplementary file 1 — Table S1. Correlation of HOXB-AS3 expression with clinical characteristics and frequent somatic gene mutations in de novo AML patients. Table S2. Correlation of HOXB-AS3 expression with clinical characteristics in MDS patients. Table S3. Correlation of HOXB-AS3 expression with frequent gene mutations in MDS patients. Figure S1. Overview of Hoxb5os in mouse HOXB cluster. Figure S2. Alignment of mouse Hoxb5os and human HOXB-AS3. Figure S3. HOXB-AS3 promotes S phase entering in the cell cycle regulation. Figure S4. GSEA pathway analysis of the differentially expressed genes in HOXB-AS3 knockdown cells compared to the control cells. Figure S5. Cell cycle pathway from Wikipathway analysis of downstream pathways affected by downregulation of HOXB-AS3 in the myeloid cell lines. Figure S6. DNA replication pathway from Wikipathway analysis of downstream pathways affected by downregulation of HOXB-AS3 in the myeloid cell lines. Figure S7. RB pathway from Wikipathway analysis of downstream pathways affected by downregulation of HOXB-AS3 in the myeloid cell lines. Figure S8. G1-S pathway from Wikipathway analysis of downstream pathways affected by downregulation of HOXB-AS3 in the myeloid cell lines. Figure S9. Quantitative PCR analysis of the expressions of indicated genes in TF-1 cells infected with lentivirus carrying pAS5.1w-Pbsd (control), or pAS5.1w-Pbsd-HOXB-AS3 (HOXB-AS3 overexpression). Figure S10. HOXB-AS3 expressions of AML patients and health donors. Figure S11. Survival analysis of AML patients stratified by the expressions of HOXB-AS3. Figure S12. HOXB-AS3 expression of non-APL AML patients and health donors. Figure S13. Survival analysis of non-APL AML patients stratified by the expressions of HOXB-AS3 in the NTUH AML cohort. Figure S14. HOXB-AS3 expressions of MDS patients and health donors. Figure S15. Overall survival of MDS patients stratified by the expressions of HOXB-AS3. Figure S16. Quantitative PCR analysis of the expressions of different variants in TF- [file 12885_2019_5822_MOESM1_ESM.pdf]

Supplementary Data

Table of Contents

	Page
**Supplementary Methods**	**1**
**Table S1.** Correlation of *HOXB-AS3* expression with clinical characteristics and frequent somatic gene mutations in *de novo* AML patients	**6**
**Table S2.** Correlation of *HOXB-AS3* expression with clinical characteristics in MDS patients	**7**
**Table S3.** Correlation of *HOXB-AS3* expression with frequent gene mutations in MDS patients	**9**
**Figure S1.** Overview of *Hoxb5os* in mouse HOXB cluster.	**10**
**Figure S2.** Alignment of mouse *Hoxb5os* and human *HOXB-AS3*.	**11**
**Figure S3.** *HOXB-AS3* promotes S phase entering in the cell cycle regulation.	**12**
**Figure S4.** GSEA pathway analysis of the differentially expressed genes in *HOXB-AS3* knockdown cells compared to the control cells.	**13**
**Figure S5.** Cell cycle pathway from Wikipathway analysis of downstream pathways affected by downregulation of *HOXB-AS3* in the myeloid cell lines.	**14**
**Figure S6.** DNA replication pathway from Wikipathway analysis of downstream pathways affected by downregulation of *HOXB-AS3* in the myeloid cell lines.	**15**
**Figure S7.** RB pathway from Wikipathway analysis of downstream pathways affected by downregulation of *HOXB-AS3* in the myeloid cell lines.	**16**
**Figure S8.** G1-S pathway from Wikipathway analysis of downstream pathways affected by downregulation of *HOXB-AS3* in the myeloid cell lines.	**17**
**Figure S9.** Quantitative PCR analysis of the expressions of indicated genes in TF-1 cells infected with lentivirus carrying pAS5.1w-Pbsd (control), or pAS5.1w-Pbsd-HOXB-AS3 (*HOXB-AS3* overexpression).	**18**
**Figure S10.** *HOXB-AS3* expressions of AML patients and health donors.	**19**
**Figure S11.** Survival analysis of AML patients stratified by the expressions of *HOXB-AS3*.	**20**
**Figure S12.** *HOXB-AS3* expression of non-APL AML patients and health donors.	**21**
**Figure S13.** Survival analysis of non-APL AML patients stratified by the expressions of *HOXB-AS3* in the NTUH AML cohort.	**22**
**Figure S14.** *HOXB-AS3* expressions of MDS patients and health donors.	**23**
**Figure S15.** Overall survival of MDS patients stratified by the expressions of *HOXB-AS3*.	**24**
**Figure S16.** Quantitative PCR analysis of the expressions of different variants in TF-1 and OCI/AML3 cell lines.	**25**
**Supplementary References**	**26**

Supplementary Methods

Microarray experiments

Five microgram total RNA per sample was purified by TRIzol or Nucleospin© RNA purification kit (Macherey-Nagel GmbH & Co. KG, Germany) with genomic DNA digestion. The RNA was sent to the National Health Research Institutes, Taiwan, and Affymetrix Gene Expression Service Lab, Academia Sinica, Taiwan, for microarray experiments. RNA concentrations and qualities were checked by Bioanalyzer. Total RNA was used for cDNA synthesis, and labeled by *in vitro* transcription following the manufacturer's instructions of Affymetrix GeneChip® Human Transcriptome Array 2.0 (HTA 2.0). After labeling, cDNA was hybridized for 16 hours at 45℃ on GeneChip® Human Transcriptome Array 2.0 in Hybridization Oven 645. GeneChips® were washed and stained in the Affymetrix Fluidics Station 450. GeneChips® were scanned by using Affymetrix GeneChip Command Console installed with a GeneChip® Scanner 7G.

Microarray data analysis

The raw data were normalized with Robust Multichip Analysis (RMA) algorithm by Affymetrix® Expression Console™ Software. Further analysis of microarrays was performed with Affymetrix® Transcriptome Analysis Console (TAC) software (http://www.affymetrix.com/estore/catalog/prod760001/AFFY/Transcriptome+Analysis+Console+%28TAC%29+Software#1_1), BRB-Array Tools v4.5.0 stable (provide by National Cancer Institute, National Institutes of Health; https://brb.nci.nih.gov/BRB-ArrayTools/), and Gene Set Enrichment Analysis (GSEA) software for pathway analysis (http://software.broadinstitute.org/gsea/index.jsp). [1, 2]

Cell cultures

OCI/AML3 and TF-1 were myeloid cell lines. TF-1 cell line was purchased from Bioresource Collection and Research Center, Hsinchu, Taiwan. OCI/AML3 was a gift from Dr. Minden (Ontario Cancer Institute/Princess Margaret Hospital, Canada). TF-1 was cultured in RPMI 1640 medium (ATCC Modification, Gibco®) with 10% heat-inactivated fetal bovine serum (FBS), 100 units per milliliter penicillin, 100 micrograms per milliliter streptomycin (Gibco®), and 4 nanograms per milliliter GM-CSF (GM-CSF Recombinant Human Protein, Gibco® PHC2011). OCI/AML3 was cultured in RPMI 1640 medium (ATCC Modification, Gibco®) with 100 units per milliliter penicillin, 100 microgram per

milliliter streptomycin (Gibco®), and 10% heat-inactivated FBS.

Constructions of lentiviral vectors with shRNA and lncRNA, and lentiviral production

RNAi reagents and vectors (pLKO_AS1010 for shRNA, pLAS5w.Pbsd for overexpression of lncRNAs, the packaging plasmid pCMV-ΔR8.91 and the envelope plasmid pMD.G) were purchased from the National Core Facility for Manipulation of Gene Function by RNAi, miRNA, miRNA sponges, and CRISPR/Genomic Research Center, Academia Sinica, Taiwan, supported by the National Core Facility Program for Biotechnology Grants of MOST (MOST 104-2319-B-001-001; also known as, "RNAi Core Lab, Academia Sinica, Taiwan"). The designs and constructs of shRNA and cDNA were performed according to the protocols provided by RNAi Core Lab, Academia Sinica, Taiwan.

Briefly, human *HOXB-AS3* variant 2 (GenBank accession code: NR_033202.2) was PCR amplified and cloned into pLAS5w.Pbsd between *Nhe*I and *Pme*I sites. *HOXB-AS3* shRNAs were designed according to the algorithm of The RNAi Consortium shRNA Library, Broad Institute. The sense and antisense oligonucleotides were annealed and ligated into pLKO_AS1010 digested by *Age*I and *Eco*RI. Because most variants of *HOXB-AS3* contain exon 4 and 6, we designed two shRNAs to target each of these two exons (Figure 1a). The target sequences of shHOXB-AS3 and shLacZ were as follows; shHOXB-AS3#1: GGACCCACCAACCAAGGAGCT (targeting exon 4 of *HOXB-AS3*), shHOXB-AS3#2: GGCACTCACTTGCAGACATCA (targeting exon 6 of *HOXB-AS3*), and shLacZ: CGCGATCGTAATCACCCGAGT.

The lentiviral production was performed according to the protocol of RNAi Core Lab, Academia Sinica, Taiwan. The lentivirus was concentrated with Lentix-X solution (Clontech©) according to the manufacturer's protocol. The concentrated lentiviral particles were suspended in DMEM with 20% heat-inactivated FBS, 100 units per milliliter penicillin, and 100 micrograms per milliliter streptomycin (Gibco®).

Lentivirus infection to generate stable cell lines

The concentrated lentiviral solution was mixed with suspension cells and polybrene with a final concentration of 8 micrograms per milliliter. The mixture of lentivirus and suspension cells were incubated at 37℃ for 24 hours. Then, the cells were centrifuged at 1000 rpm for 5 minutes at room temperature. The supernatant with lentivirus was removed, and the suspension cells were

refreshed with the medium used to maintain the cell lines. Two days later, blasticidin or puromycin was added in the medium for drug selection. After seven days of drug selection, the survived cells were used for validation and further experiments.

Quantitative real time PCR

Total RNA was isolated from cells by Nucleospin® RNA kit (Macherey-Nagel GmbH & Co. KG, Germany). Two micrograms RNA was used to synthesize complementary DNA (cDNA) by iScript™ cDNA Synthesis Kit (Bio-Rad Laboratories, Inc). LightCycler® 480 SYBR Green I Master (Roche) was used for quantitative real time PCR. Quantitative real time PCR was performed with Roche LightCycler 480 II. The annealing temperature was 60℃. The primers were as the follows.

β2 microglobulin forward: 5'-AGCAGCATCATGGAGGTTTG-3';
β2 microglobulin reverse: 5'-AGCCCTCCTAGAGCTACCTG-3';
18s rRNA forward: 5'-ACGAAAGTCGGAGGTTCGAA-3';
18s rRNA reverse: 5'-GTGTTGAGTCAAATTAAGCCGCAG-3';
GAPDH forward: 5’-GTCAGTGGTGGACCTGACCT-3';
GAPDH reverse: 5'-ATACCAGGAAATGAGCTTGACAAAG-3';
MALAT1 forward: 5’-GAATTGCGTCATTTAAAGCCTAGTT -3’;
MALAT1 reverse: 5’-GTTTCATCCTACCACTCCCAATTAAT-3’;
NEAT1 forward: 5’-TCGGGTATGCTGTTGTGAAA-3’;
NEAT1 reverse: 5’-TGACGTAACAGAATTAGTTCTTACCA-3’;
HOXB-AS3 variant 2/3/5 forward: 5'-ACAACCGAGTGGAACTGACG-3';
HOXB-AS3 variant 2/3/5 reverse: 5'-AAGCCAAGGCTGTTCAAGGT-3' (targeting exon 6);
HOXB-AS3 variant 1/4 forward: 5'-GGCCCTATAGAAACCAGGACG-3';
HOXB-AS3 variant 1/4 reverse: 5'-GAAGGGAATGAGTCCGGGAG-3';
MCM3 forward: 5'-TCTGGGACCTTCAGGACTGT-3';
MCM3 reverse: 5'-TTGATGTCCCCACGGATGTG-3';
MCM4 forward: 5'-GATTCCCGAGATGCAGGAGG-3';
MCM4 reverse: 5'-GCACAGCTCGATAGATGCCT-3';
MCM6 forward: 5'-TCCAAGCTTAGCACACCAGG-3';
MCM6 reverse: 5'-GAACGCCACCAAAGAGCATC-3';
PCNA forward: 5'-GGCCGAAGATAACGCGGATA-3';
PCNA reverse: 5'-TGTCACCGTTGAAGAGAGTGG-3';
CKD1 forward: 5'-ACAGGTCAAGTGGTAGCCAT-3';
CKD1 reverse: 5'-CCATGTACTGACCAGGAGGG-3';

CCNB2 forward: 5'-CACTCTTGCCTTCCCCGTC-3';
CCNB2 reverse: 5'-AGCTACTTGTGCTGCTCTGG-3';
CDC25A forward: 5'-GCAACCACTGGAGGTGAAGA-3';
CDC25A reverse: 5'-TCATCTGGGTCGATGAGCTG-3'.

Proliferation assay

After OCI/AML3 cells were infected with lentivirus containing shRNA for one night, the cells were refreshed with the culture medium for two days. On the fourth day, the GFP-positive cells were sorted for proliferation assay by BD FACSJazz at the Flow Cytometry Core Facility of the institute of Biomedical Sciences, Academia Sinica. On the Day 0, 1×10^6 GFP-positive cells were seeded in 5 mL culture medium per flask, and three flasks per group. The cell numbers were counted for six consecutive days.

BrdU flow assay

BD Pharmingen™ APC BrdU Flow Kits (Cat. NO. 552598) was used for BrdU cell proliferation assay. Cells were incubated with 10 μM BrdU at 37℃ for three hours, and then harvested for BrdU flow assay. The detailed method was described in the user manual of the manufacture. The flow cytometry was performed on LSR II (BD Bioscience, San Jose, CA) through the service provided by the Flow Cytometric Analyzing and Sorting Core Facility at the NTUH, and on FACS Canto II (BD Bioscience, San Jose, CA) through the service provided by the Molecular and Immune Function Laboratory at Tai Cheng Stem Cell Therapy Center at the National Taiwan University.

Nuclear-cytoplasm fractionation

The detailed method of nuclear-cytoplasm fractionation for RNA was described previously.[3] Briefly, 1×10^6 to 1×10^7 cells were harvested and washed with PBS twice. Then, the cells were resuspended in 1 milliliter RSB (10 mM Tris, pH 7.4; 10 mM NaCl; 3mM $MgCl_2$), incubated on ice for three minutes, and centrifuged at 4 °C with 4000 rpm for three minutes. The cells were resuspended with four times of its volume of lysis buffer RSBG40 (10 mM Tris, pH 7.4; 10 mM NaCl, 3 mM $MgCl_2$; 10% glycerol; 0.5% Nonidet P-40; 0.5 mM dithiothreitol (DTT); and 100 units per milliliter rRNasin (Promega,WI)) by slow pipetting. The cells were centrifuged at 7000 rpm for three minutes. The supernatant was collected as the cytoplasmic fraction. The pellet was re-suspended with RSBG40, supplemented with one-tenth volume of detergent (3.3% (wt/wt) sodium deoxycholate and 6.6% (vol/vol) Tween 40) with slow

vortex, incubated on ice for five minutes, and centrifuged at 7000 rpm for three minutes to pellet nuclei again. The supernatant was pooled with the aforementioned cytoplasmic fraction. The nuclear pellet was washed with RSBG40 again, and centrifuged at 10,000 rpm for five minutes. The status of cell lysis was checked by the microscope. The nuclear pellet was resuspended in 250 microliters of lysis buffer RSBG40 and 1 milliliter TRIzol was added. The 250 microliters of cytoplasmic fraction was mixed with 1 milliliter TRIzol by vortex. The mixtures with TRIzol were incubated at the room temperature for five to ten minutes. RNA was extracted with TRIzol according to the manufacturer's instructions.

Table S1. Correlation of *HOXB-AS3* expression with clinical characteristics and frequent somatic gene mutations in *de novo* AML patients

	Total	*HOXB-AS3* expression		P value
		Higher	Lower	
**Patient number (n)**	193	96	97	
**Sex (n, %)**				
Male		42 (43.7%)	60 (61.9%)	0.0120*
Female		54 (56.2%)	37 (38.1%)	
**Age (years; median, range)**	46.7 (16.1 – 82.7)	52.7 (16.1 – 76.8)	42.8 (18.2 – 82.7)	0.0010*
**Laboratory (median, range)**				
WBC (k/μL)	27.770 (0.380 – 423.000)	35.865 (0.380 – 423.000)	23.530 (0.470-380.180)	0.0684
Hb (g/dL)	7.9 (3.3 – 13.0)	7.7 (3.7 – 12.1)	8.1 (3.3 – 13.0)	0.0946
PLT (k/μL)	44.0 (2.0 – 493.0)	52.5 (5.0 – 412.0)	38.0 (2.0 – 493.0)	0.0449*
LDH (U/L)	1019 (280 – 8280)	924 (280 – 7734)	1036 (286 – 8280)	0.4282
Bone marrow blasts (%)	63.0 (16.0 – 98.5)	61.3 (20.5 – 97.5)	65.5 (16.0 – 98.5)	0.1267
Peripheral blood blasts (%)	53.6 (0.0 – 99.0)	55.0 (1.0 – 99.0)	51.8 (0.0 – 95.0)	0.3927
**Cytogenetic risk groups (n, %)[#]**				< 0.0001*
Favorable	44 (22.8%)	4 (4.2%)	40 (41.2%)	
Intermediate	119 (61.7%)	71 (74.0%)	48 (49.5%)	
Adverse	21 (10.9%)	12 (12.5%)	9 (9.3%)	
Unknown	9 (4.7%)	9 (9.4%)	0 (0.0%)	
**CR rate**	154 (79.8%)	73 (76.0%)	81 (83.5%)	0.1978
**Relapse rate**	82 (42.5%)	46 (47.9%)	36 (37.1%)	0.0306*
**Somatic gene mutations (n, %)**				
Mutated *NPM1* without *FLT3*-ITD	26 (13.5%)	25 (26.0%)	1 (1.0%)	< 0.0001*
CEBPα double mutation	25 (13.1%)	4 (4.2%)	21 (21.9%)	0.0004*
MLL/PTD	10 (5.2%)	10 (10.4%)	0 (0.0%)	0.0007*
TP53	4 (2.2%)	4 (4.3%)	0 (0.0%)	0.0591
RUNX1	22 (11.4%)	17 (17.7%)	5 (5.2%)	0.0062*

* P value < 0.05.

Abbreviations: WBC, white blood cell count; Hb, hemoglobin; PLT, platelet count; LDH, lactic dehydrogenase; CR, complete remission; *MLL*/PTD, partial tandem duplication of *MLL* gene.

Table S2. Correlation of *HOXB-AS3* expression with clinical characteristics in MDS patients

	Total	*HOXB-AS3* expression		P value
		Higher	Lower	
**Patient number (n)**	157	39	118	
**Sex (n, %)**				
Male	107 (68.2%)	31 (79.5%)	76 (64.4%)	0.0807
Female	50 (31.8%)	8 (20.5%)	42 (35.6%)	
**Age (years; median, range)**	69 (19 – 94)	71 (29 – 89)	69 (19 – 94)	0.5209
**Laboratory (median, range)**				
WBC (k/μL)	3.700 (0.490 – 20.440)	3.830 (0.490-20.440)	3.700 (1.210 – 15.010)	0.8892
Hb (g/dL)	8.2 (3.5 – 14.6)	8.7 (5.6 – 13.0)	8.0 (3.5 – 14.6)	0.0186*
PLT (k/μL)	85.0 (3.0 – 721.0)	84.0 (9.0 – 721.0)	87.5 (3.0 – 607.0)	0.8374
LDH (U/L)	460 (145 – 2695)	527 (230 – 1481)	453 (145 – 2695)	0.2909
Bone marrow blasts (%)	3 (0 - 18.8)	9 (0.8 – 18.8)	2 (0 – 17.2)	< 0.0001*
Peripheral blood blasts (%)	0 (0 – 16)	0 (0 – 16.0)	0 (0 – 13.0)	0.1037
**IPSS risk group (n, %)#**				0.1513
Low	31 (20.3%)	5 (13.2%)	26 (22.6%)	
Intermediate-1	72 (47.1%)	15 (39.5%)	57 (49.6%)	
Intermediate-2	35 (22.9%)	13 (34.2%)	22 (19.1%)	
High	15 (9.8%)	5 (13.2%)	10 (8.7%)	
**WHO 2016 classification (n, %)**				0.0077*
MDS with del(5q)	3 (1.9%)	0	3 (2.5%)	
SLD	35 (22.3%)	4 (10.3%)	31 (26.3%)	
MLD	32 (20.4%)	5 (12.8%)	27 (22.9%)	
RS-SLD	11 (7.0%)	3 (7.7%)	8 (6.8%)	
RS-MLD	7 (4.5%)	0	7 (5.9%)	
EB-1	29 (18.5%)	9 (23.1%)	20 (16.9%)	
EB-2	40 (25.5%)	18 (46.2%)	22 (18.6%)	
**Leukemia transformation rate (n, %)**	34 (21.7%)	14 (35.9%)	20 (16.9%)	0.0130*

* P value < 0.05.
# Only 153 patients, including 38 with high *HOXB-AS3* and 115 with low *HOXB-AS3*, had complete hemograms for IPSS calculation.
Abbreviations: WBC, white blood cell count; Hb, hemoglobin; PLT, platelet count; LDH, lactic dehydrogenase; IPSS, International Prognostic Scoring System; SLD, single lineage

dysplasia; MLD, multilineage dysplasia; RS-SLD, ring sideroblasts with single lineage dysplasia; RS-MLD, ring sideroblasts with multilineage dysplasia; EB-1, excess blasts-1; EB-2, excess blasts-2.

Table S3. Correlation of *HOXB-AS3* expression with frequent gene mutations in MDS patients

	Total	*HOXB-AS3* expression		P value
		Higher	Lower	
**Patient number (n)**	157	39	118	
***RUNX1* (n, %)**	25 (15.9%)	14 (35.9%)	11 (9.3%)	0.0001*
***ASXL1* (n, %)#**	33 (21.3%)	15 (39.5%)	18 (15.4%)	0.0017*
***DNMT3A* (n, %)**	24 (15.3%)	6 (15.4%)	18 (15.3%)	0.9844
***EZH2* (n, %)**	10 (6.4%)	5 (12.8%)	5 (4.2%)	0.0579
***IDH2* (n, %)#**	4 (2.6%)	3 (7.9%)	1 (0.9%)	0.0178*
***KRAS* (n, %)#**	1 (0.6%)	0	1 (0.8%)	0.5704
***NRAS* (n, %)**	3 (1.9%)	1 (2.6%)	2 (1.7%)	0.7319
***TP53* (n, %)**	10 (6.4%)	1 (2.6%)	9 (7.6%)	0.2632
***SETBP1* (n, %)**	4 (2.5%)	0	4 (3.4%)	0.5628
***SF3B1* (n, %)**	25 (15.9%)	5 (12.8%)	20 (16.9%)	0.5426
***SRSF2* (n, %)**	23 (14.6%)	9 (23.1%)	14 (11.9%)	0.0870
***TET2* (n, %)#**	21 (13.5%)	4 (10.3%)	17 (14.7%)	0.4889
***U2AF35* (n, %)**	12 (7.6%)	4 (10.3%)	8 (6.8%)	0.4801
***ZRSR2* (n, %)#**	15 (10.0%)	3 (7.9%)	12 (10.7%)	0.6178

* P value < 0.05, Chi-squared test

# Because of the limitation of DNA samples, not all patients had enough genomic DNA samples for analysis of these gene mutations. Thirty eight patients with high *HOXB-AS3* and 117 with low *HOXB-AS3* had genomic DNA for *ASXL1* mutation analysis, 38 with high *HOXB-AS3* and 117 with low *HOXB-AS3* for *IDH2* mutation analysis, 38 with high *HOXB-AS3* for *KRAS* mutation analysis, 116 with low *HOXB-AS3* for *TET2* mutation analysis, and 38 with high *HOXB-AS3* and 112 with low *HOXB-AS3* for *ZRSR2* mutation analysis. All other mutations were analyzed in total cohort.

Supplementary Figures

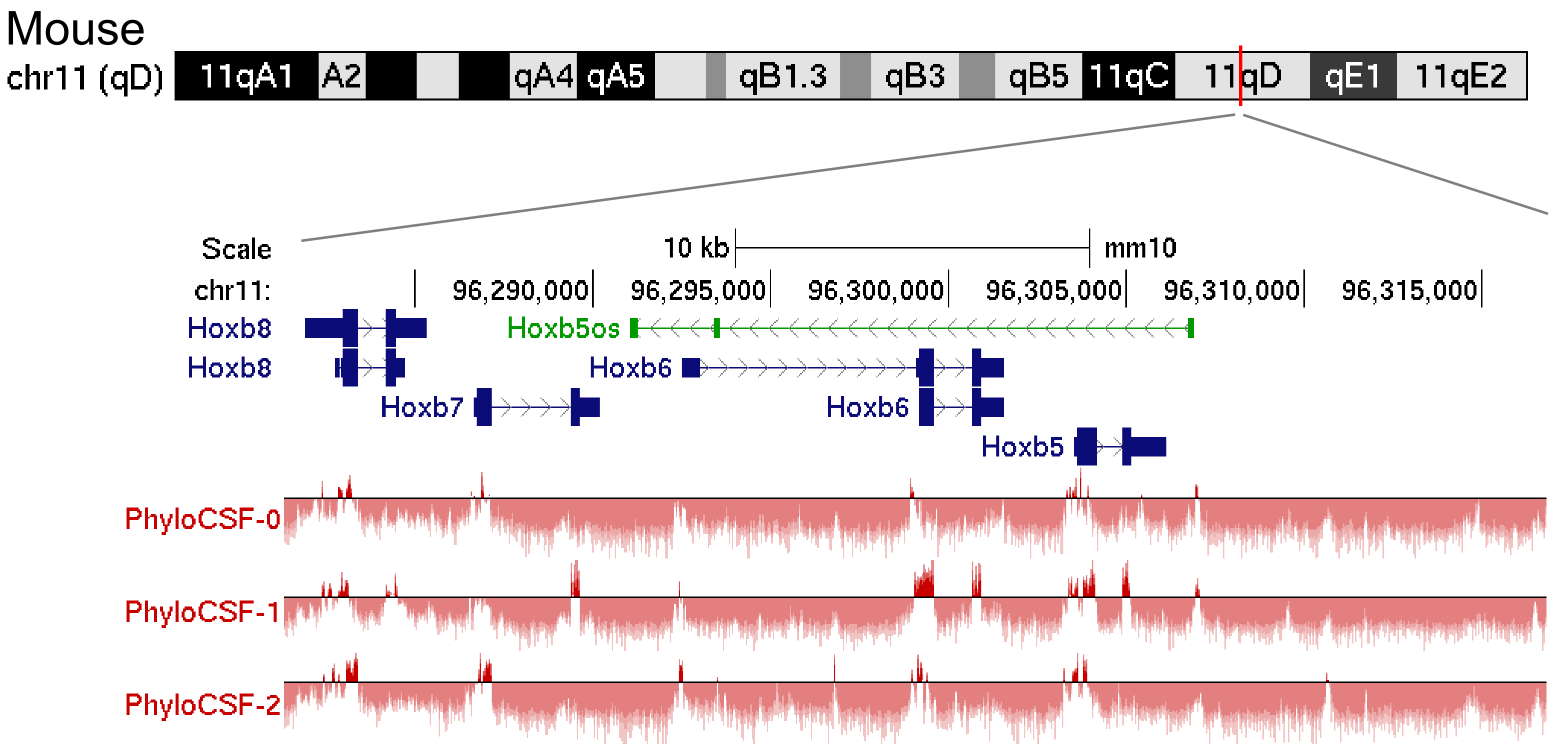

Figure S1. Overview of *Hoxb5os* in mouse HOXB cluster. Mouse HOXB cluster is located at mouse chromosome 11. An anti-sense transcript, *Hoxb5os*, is overlapped with *Hoxb5* and *Hoxb6.* The genomic data from ENCODE project are taken from UCSC genome browser.

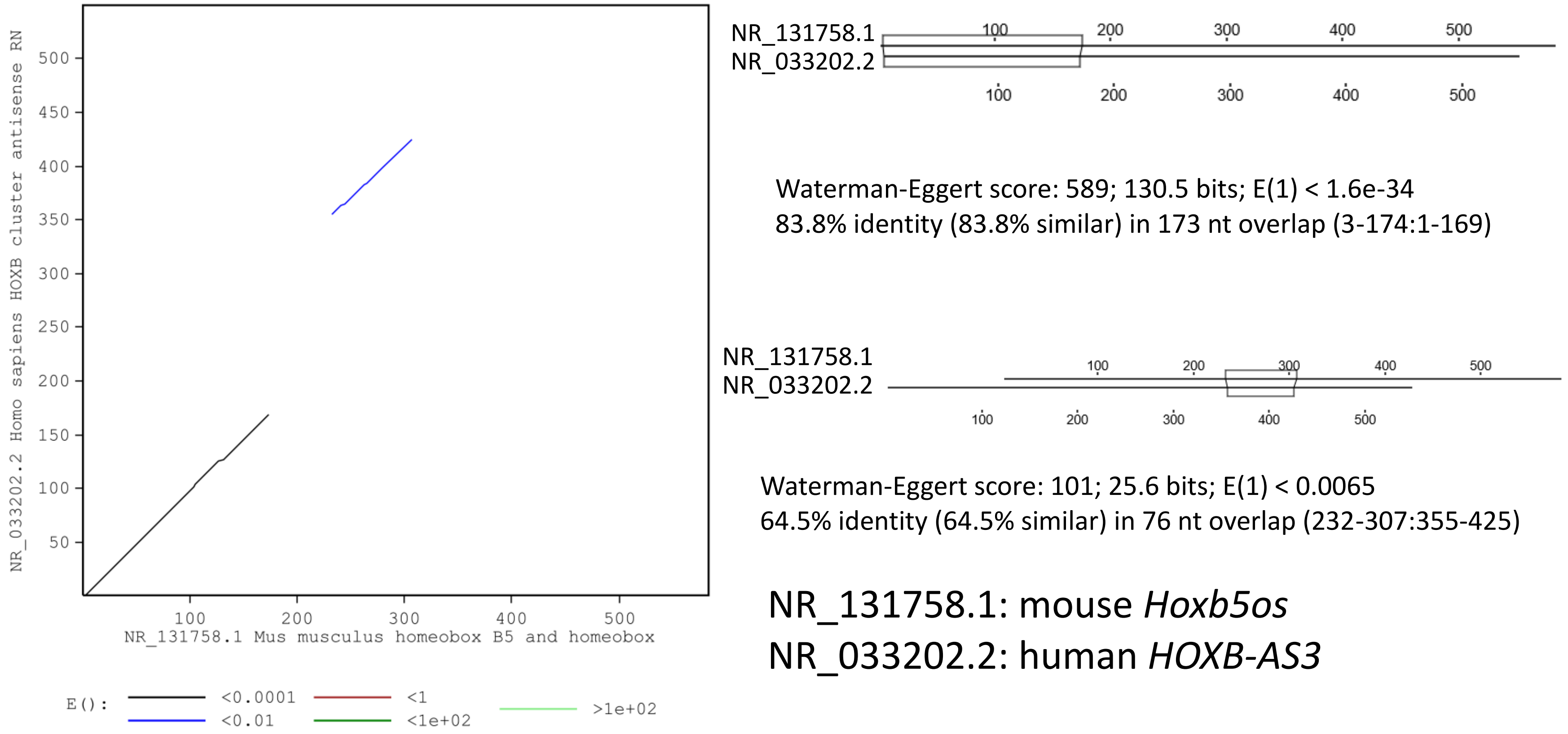

Figure S2. Alignment of mouse *Hoxb5os* and human *HOXB-AS3*. The nucleotide comparison between human *HOXB-AS3* and mouse *Hoxb5os*. NR_131758.1 is mouse Hoxb5os, and NR_033202.2 is human *HOXB-AS3*. Two conserved areas are computed and demonstrated by plalign (http://fasta.bioch.virginia.edu/).

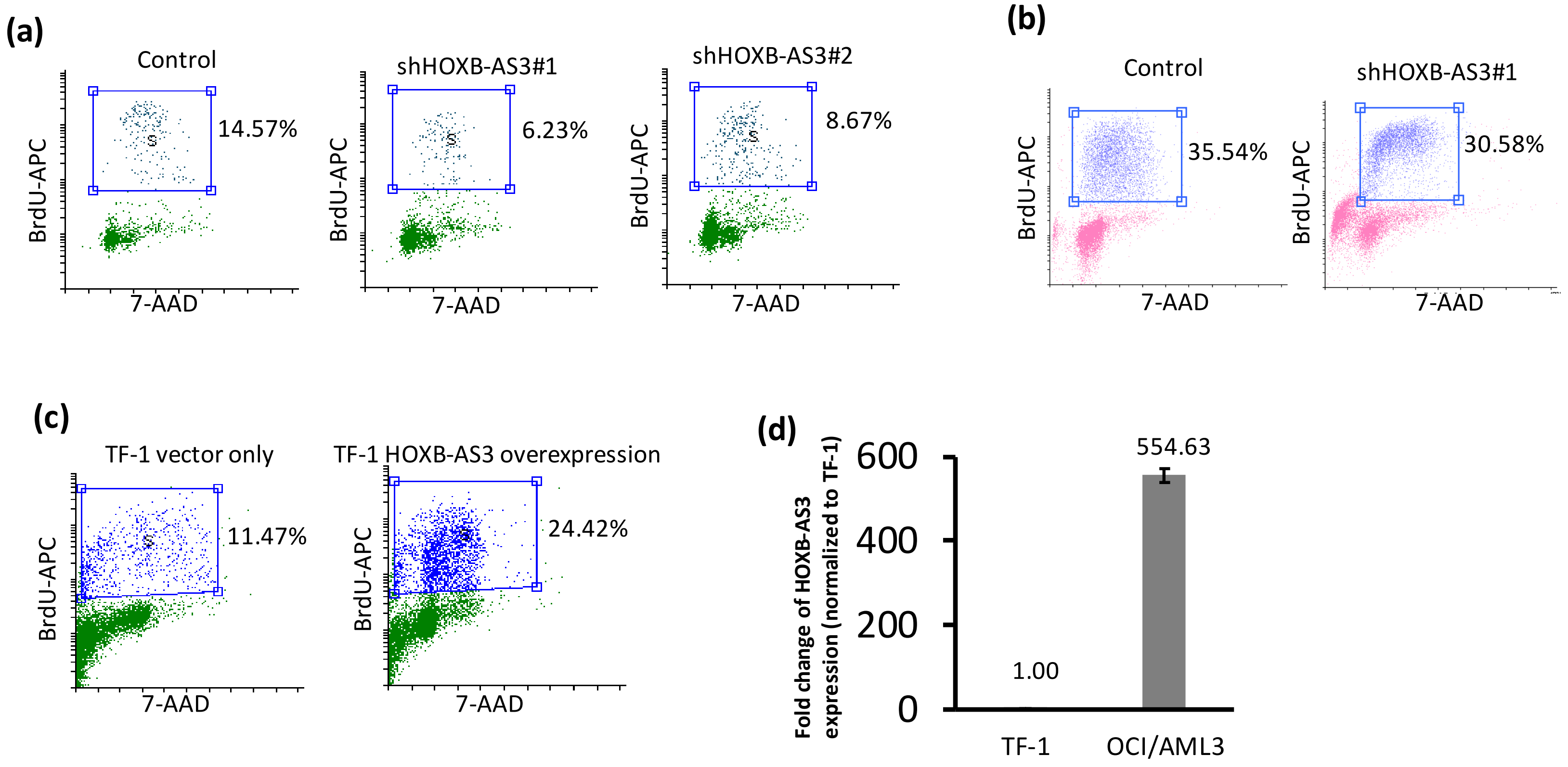

Figure S3. *HOXB-AS3* promotes S phase entering in the cell cycle regulation. (a) Representative data of BrdU flow assay of the cells shown in Figure 2c. **(b)** Representative data of BrdU flow assay of the cells shown in Figure 2e. **(c)** Representative data of BrdU flow assay of the cells shown in Figure 2g. **(d)** *HOXB-AS3* expression is higher in OCI/AML3 cells than that in TF-1 cells. (* meant that P value was less than 0.05.)

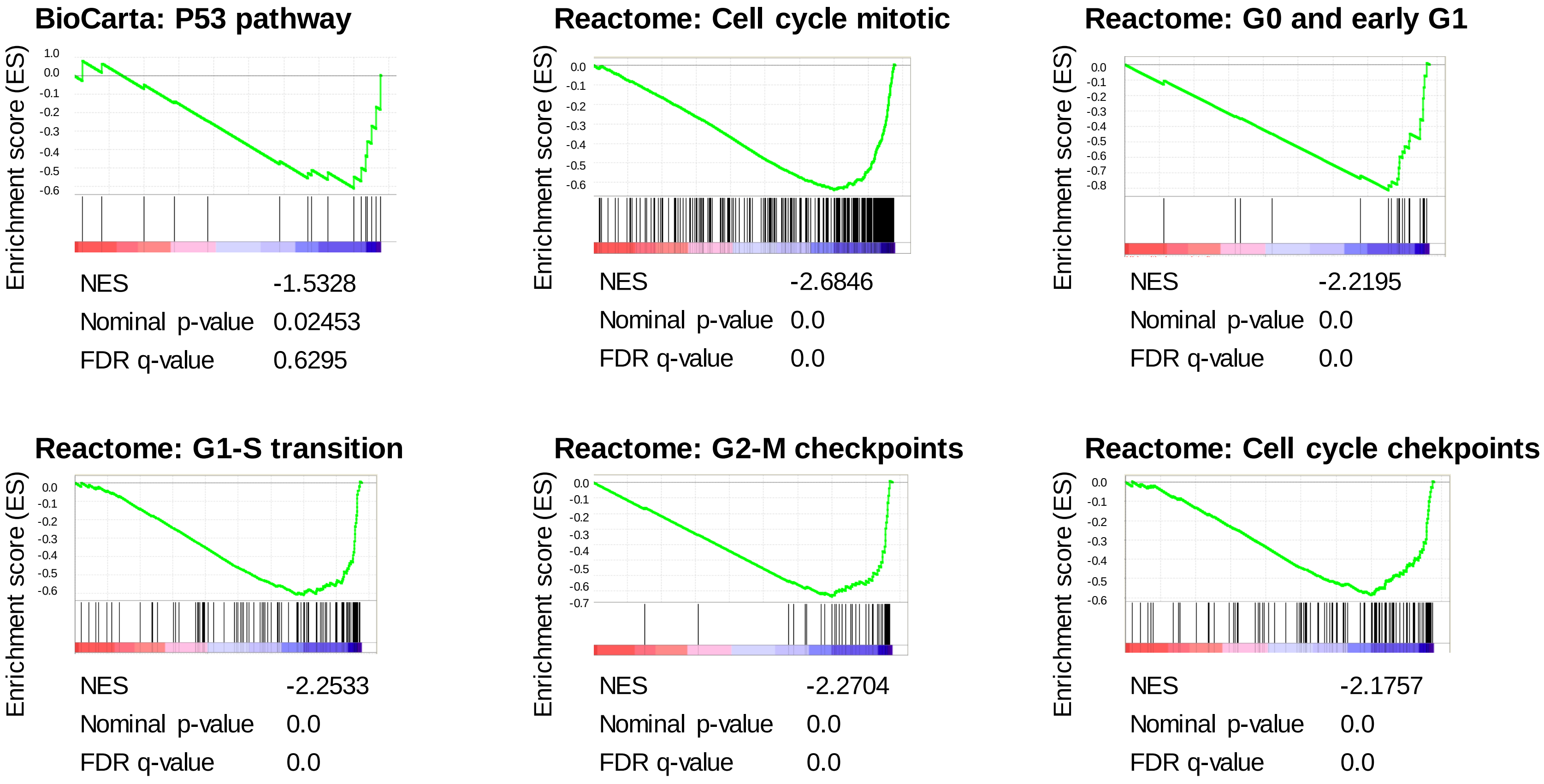

Figure S4. GSEA pathway analysis of the differentially expressed genes in *HOXB-AS3* knockdown cells compared to the control cells. All pathways in this figure were contributed by Reactome or BioCarta.

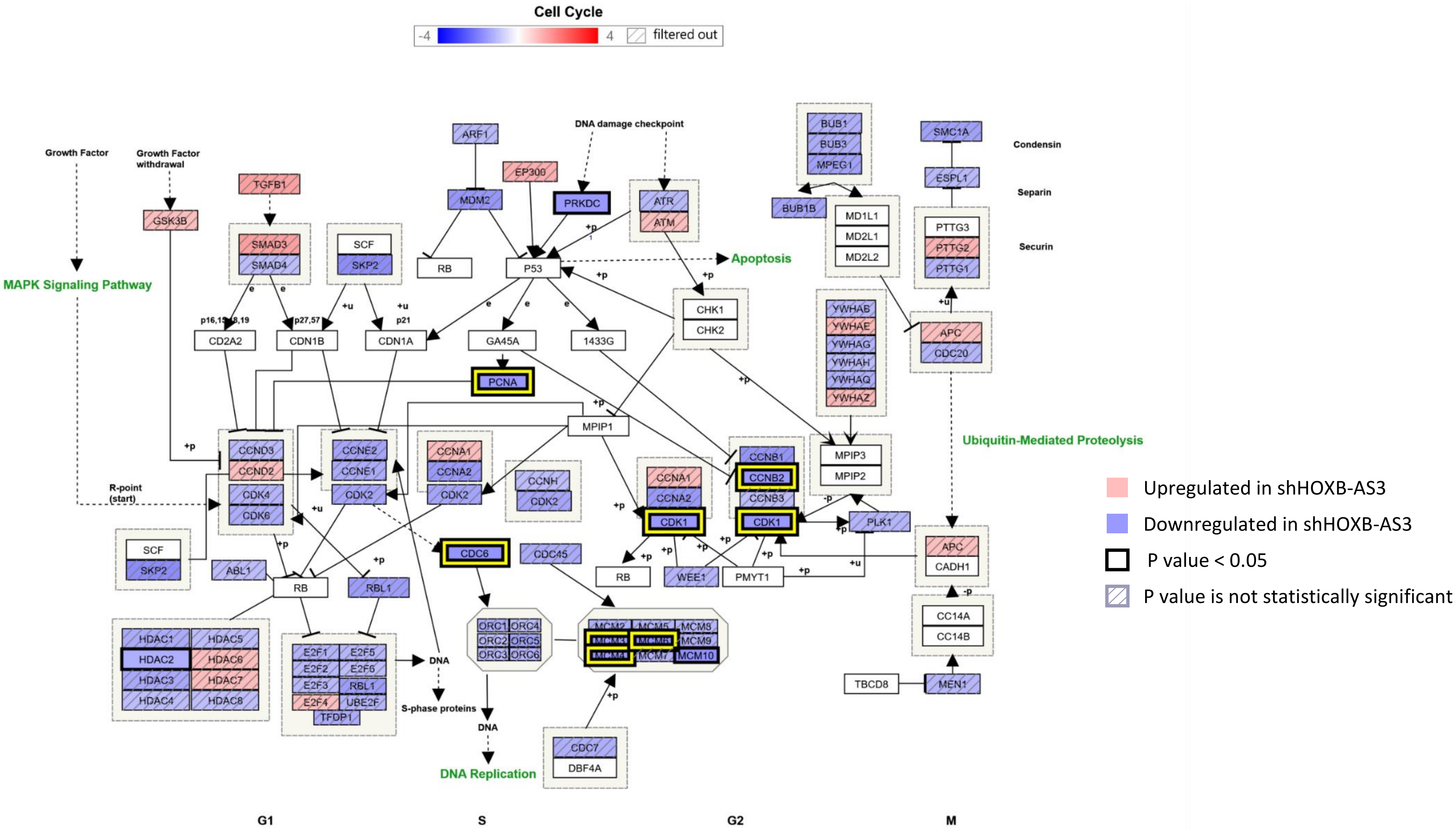

Figure S5. Cell cycle pathway from Wikipathway analysis of downstream pathways affected by downregulation of *HOXB-AS3* in the myeloid cell lines. The figure and analysis were computed by Affymetrix® TAC software.

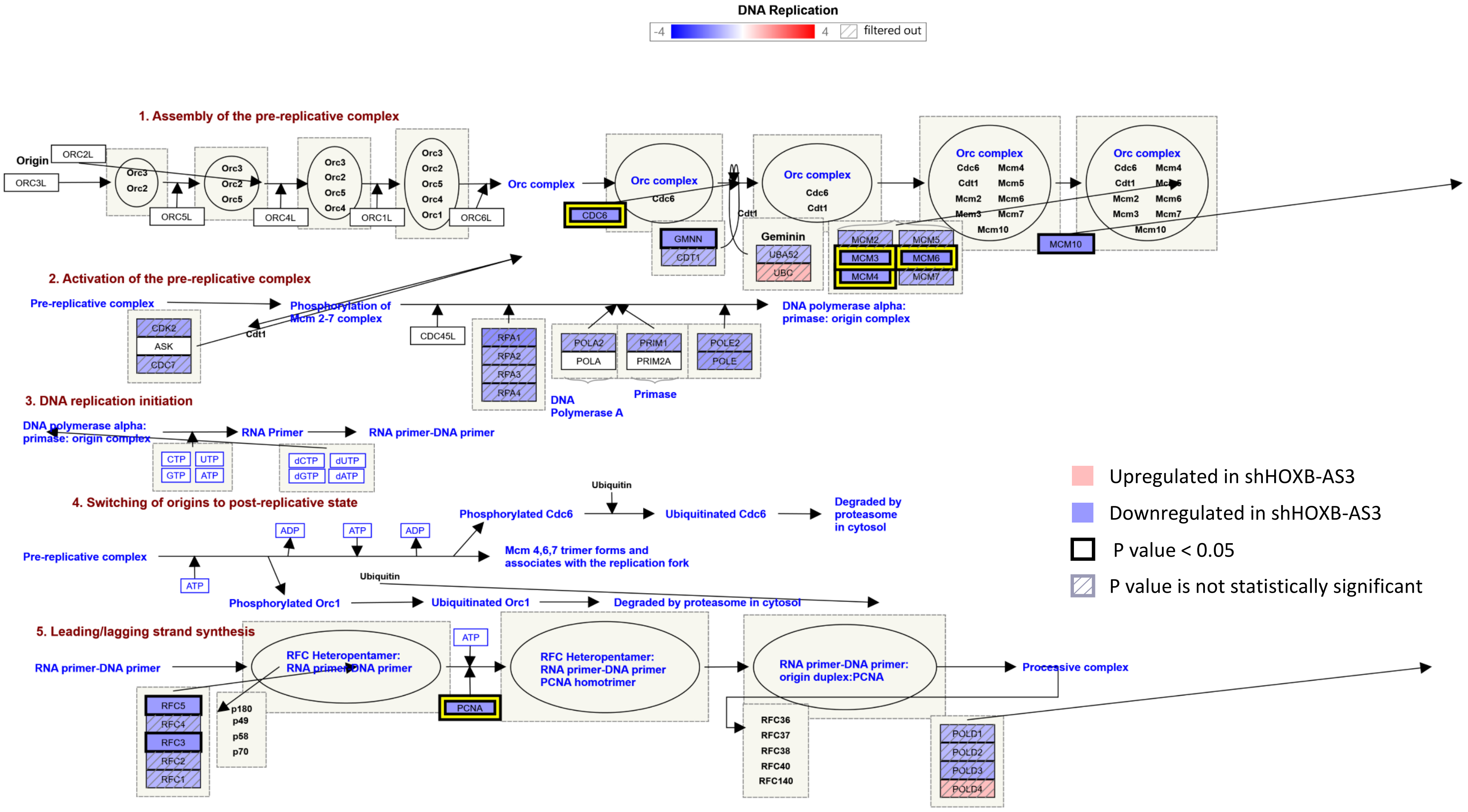

Figure S6. DNA replication pathway from Wikipathway analysis of downstream pathways affected by downregulation of *HOXB-AS3* in the myeloid cell lines. The figure and analysis were computed by Affymetrix® TAC software.

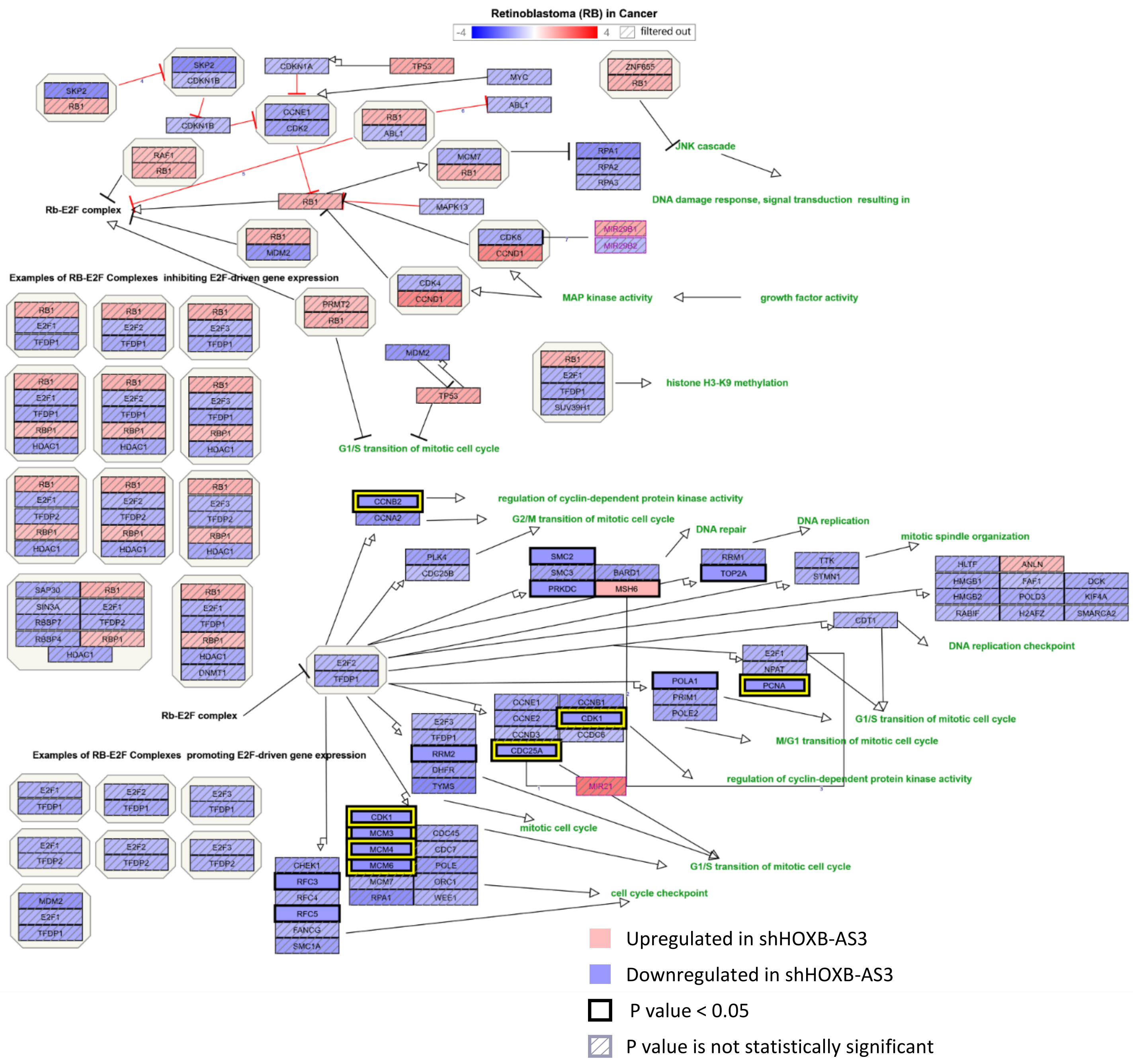

Figure S7. RB pathway from Wikipathway analysis of downstream pathways affected by downregulation of *HOXB-AS3* in the myeloid cell lines. The figure and analysis were computed by Affymetrix® TAC software.

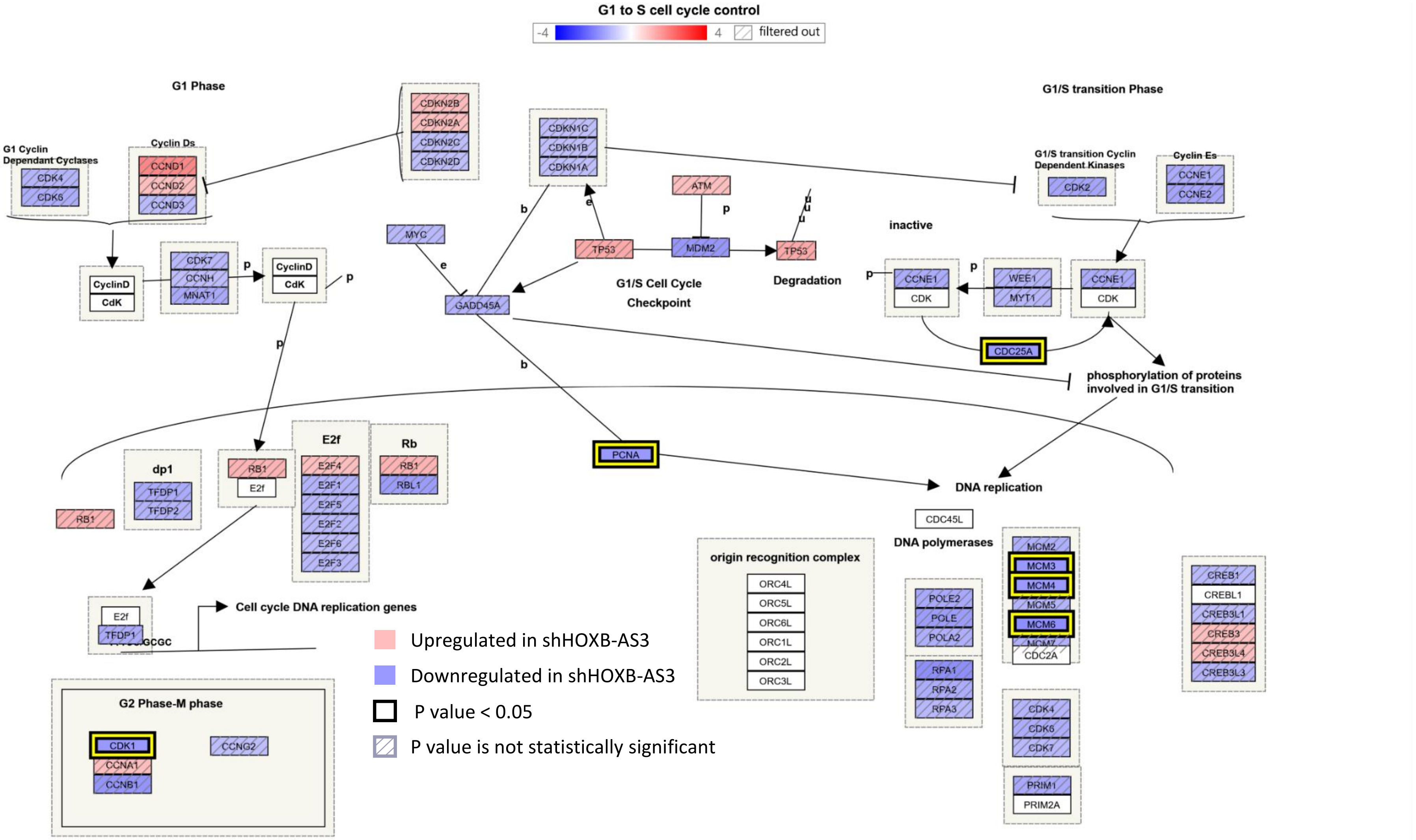

Figure S8. G1-S pathway from Wikipathway analysis of downstream pathways affected by downregulation of *HOXB-AS3* in the myeloid cell lines. The figure and analysis were computed by Affymetrix® TAC software.

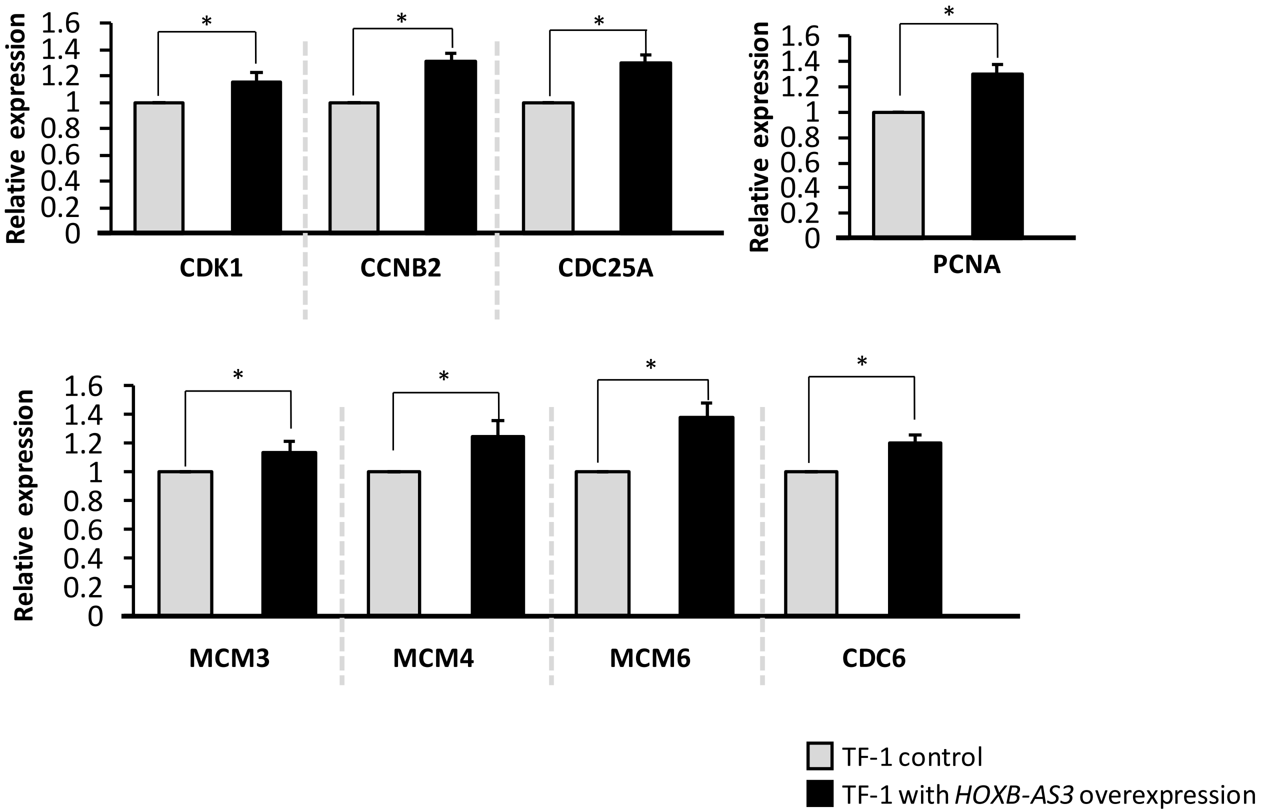

Figure S9. Quantitative PCR analysis of the expressions of indicated genes in TF-1 cells infected with lentivirus carrying pAS5.1w-Pbsd (control), or pAS5.1w-Pbsd-HOXB-AS3 (*HOXB-AS3* overexpression). Data were derived from the triple repeats of experiments. P values were calculated by Kruskal-Wallis test. (* meant that P value was less than 0.05.)

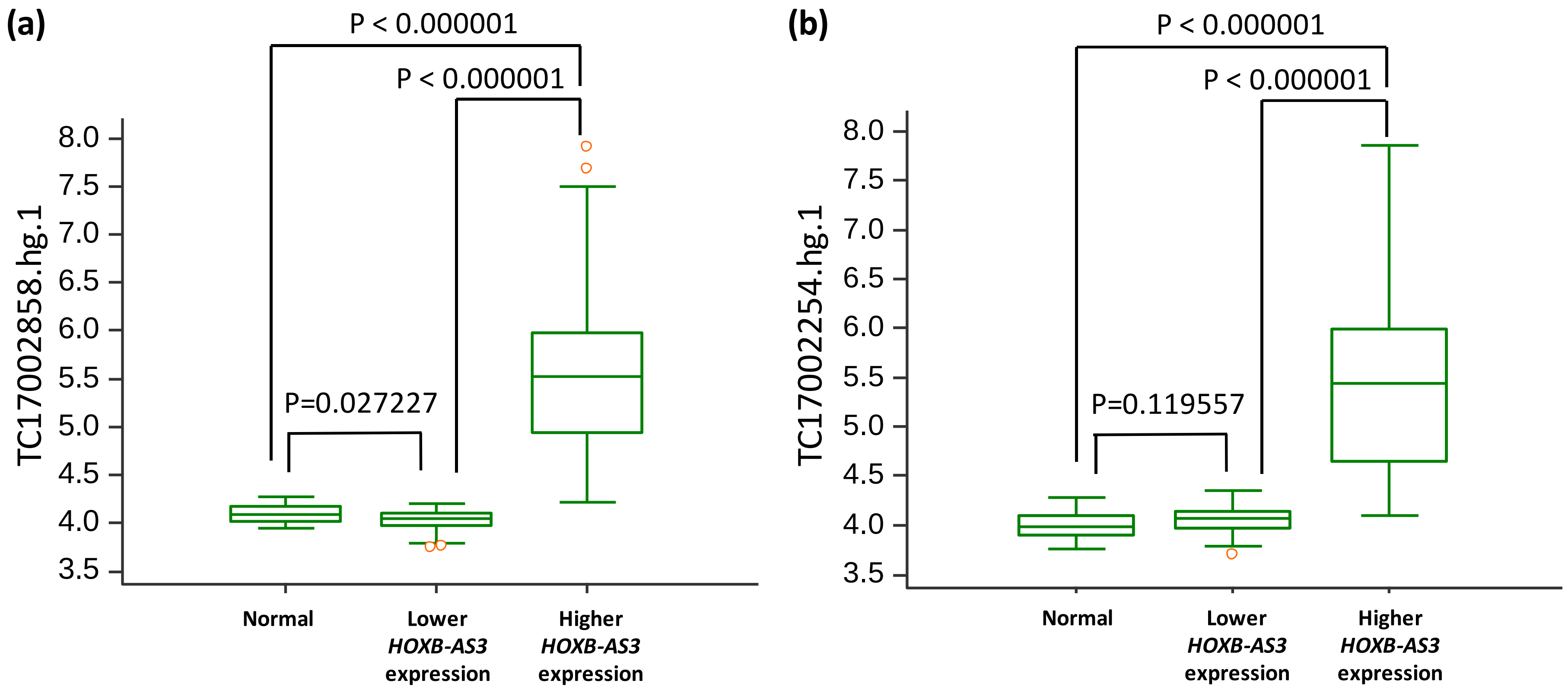

Figure S10. *HOXB-AS3* expressions of AML patients and health donors. (a) Transcript cluster: TC17002858.hg.1, and **(b)** Transcript cluster: TC17002254.hg.1 on Affymetrix GeneChip® HTA 2.0 arrays.

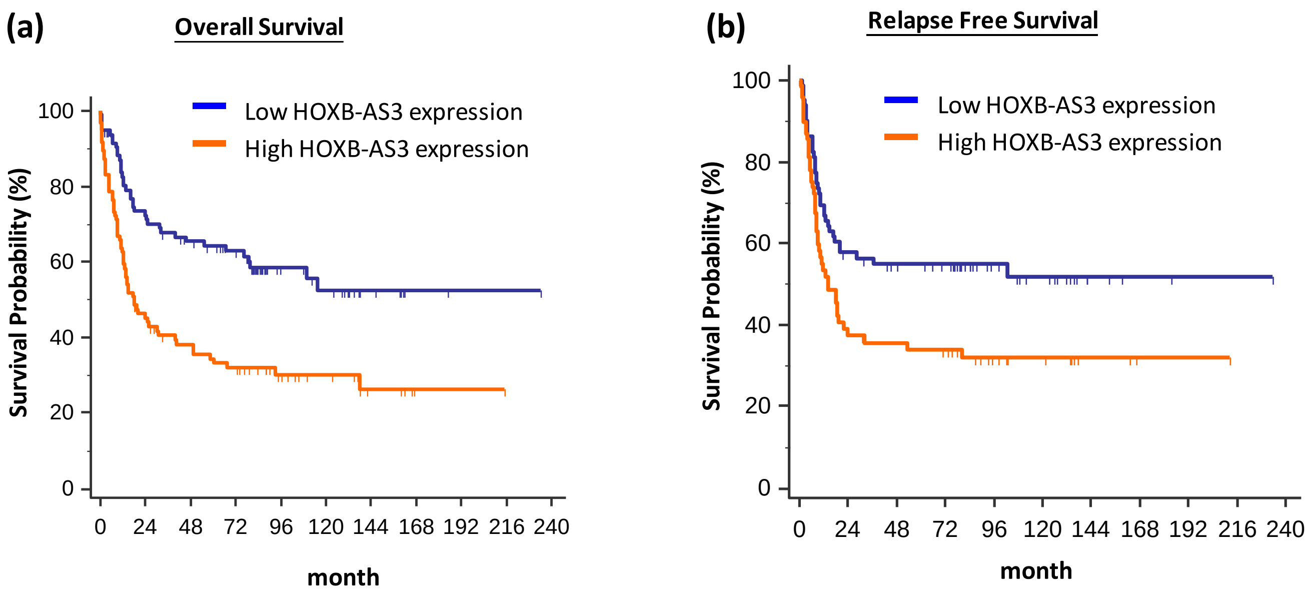

HOXB-AS3 expression	N	Median (m)	P value	HR (95% CI)
Lower	97	NR	0.0001	1
Higher	96	17.7		2.2239 (1.5029 - 3.2909)
Total patients	193	55.2		

HOXB-AS3 expression	N	Median (m)	P value	HR (95% CI)
Lower	81	NR	0.0139	1
Higher	73	142		1.7236 (1.1048 - 2.6890)
Total patients	154	20.2		

Figure S11. Survival analysis of *de novo* AML patients stratified by the expressions of *HOXB-AS3*. (a) Overall survival (OS) in the NTUH AML cohort. **(b)** Relapse free survival in the NTUH AML cohort. The patients in the NTUH AML cohort were stratified by the expressions of transcript cluster: TC17002254.hg.1 on Affymetrix GeneChip® HTA 2.0 arrays.

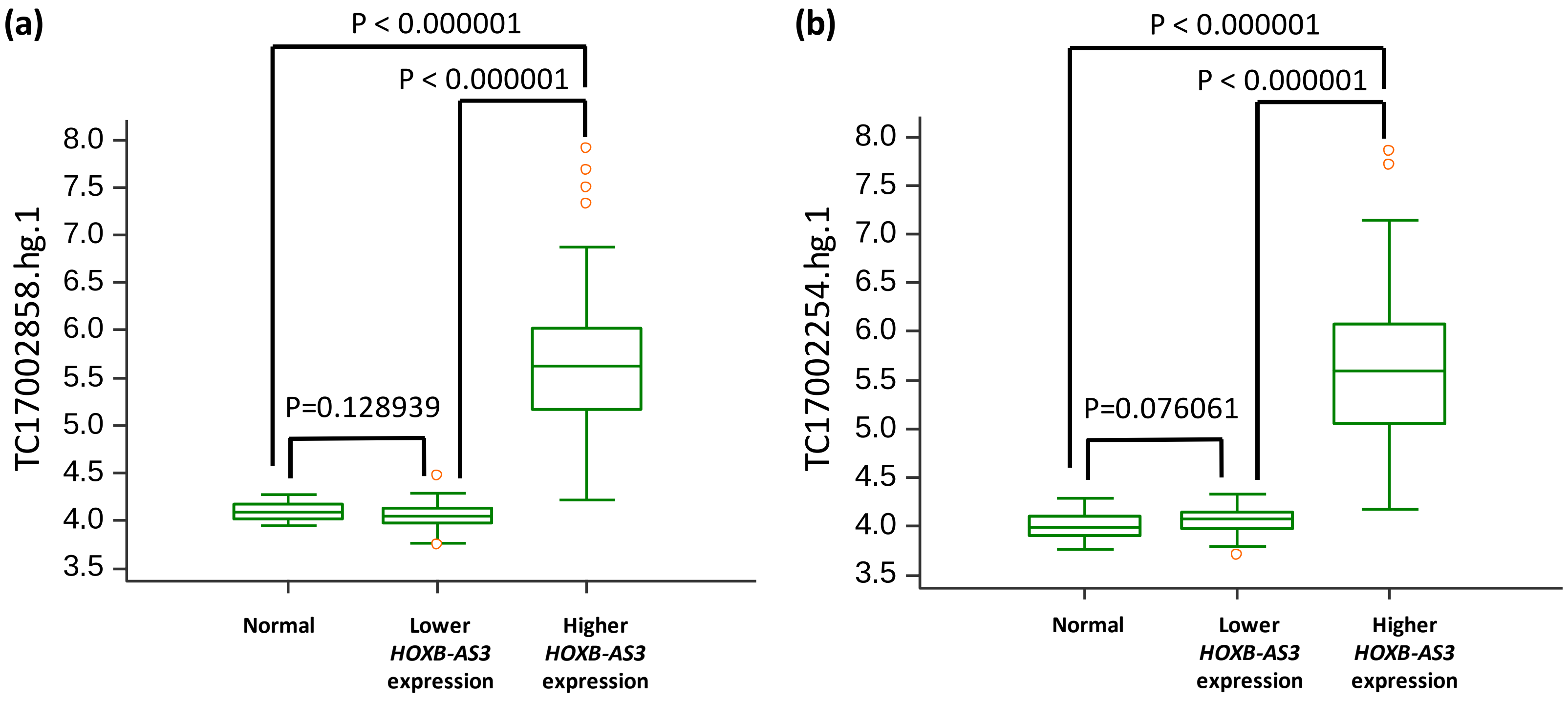

Figure S12. *HOXB-AS3* expression of non-APL AML patients and health donors. (a) Transcript cluster: TC17002858.hg.1, and **(b)** Transcript cluster: TC17002254.hg.1 on Affymetrix GeneChip® HTA 2.0 arrays.

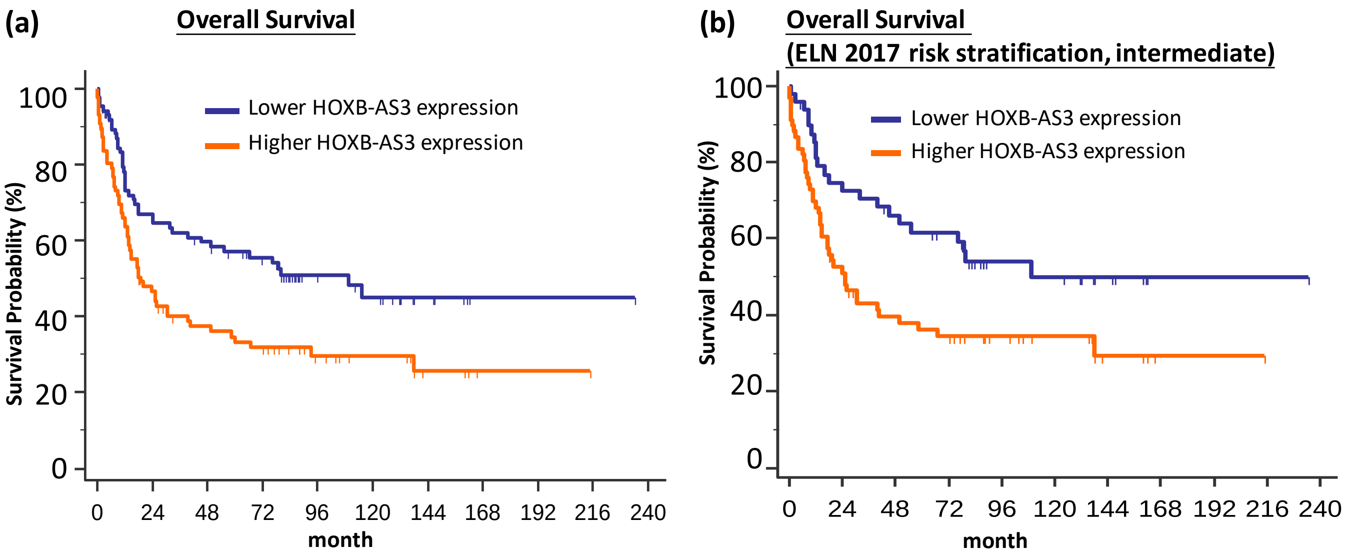

HOXB-AS3 expression	N	Median (m)	P value	HR (95% CI)
Lower	87	109.7	0.0045	1
Higher	87	19.13		1.7671 (1.1888 - 2.6268)
Total patients	174	39.7		

HOXB-AS3 expression	N	Median (m)	P value	HR (95% CI)
Lower	50	109.7	0.0121	1
Higher	68	25.1		1.9027 (1.1700 - 3.0942)
Total patients	118	49.5		

Figure S13. Survival analysis of non-APL AML patients stratified by the expressions of *HOXB-AS3* in the NTUH AML cohort. **(a)** Overall survival (OS) , **(b)** OS of the patients with intermediate risk of ELN 2017 risk stratification. The patients in the NTUH AML cohort were stratified by the expressions of transcript cluster: TC17002858.hg.1 on Affymetrix GeneChip® HTA 2.0 arrays.

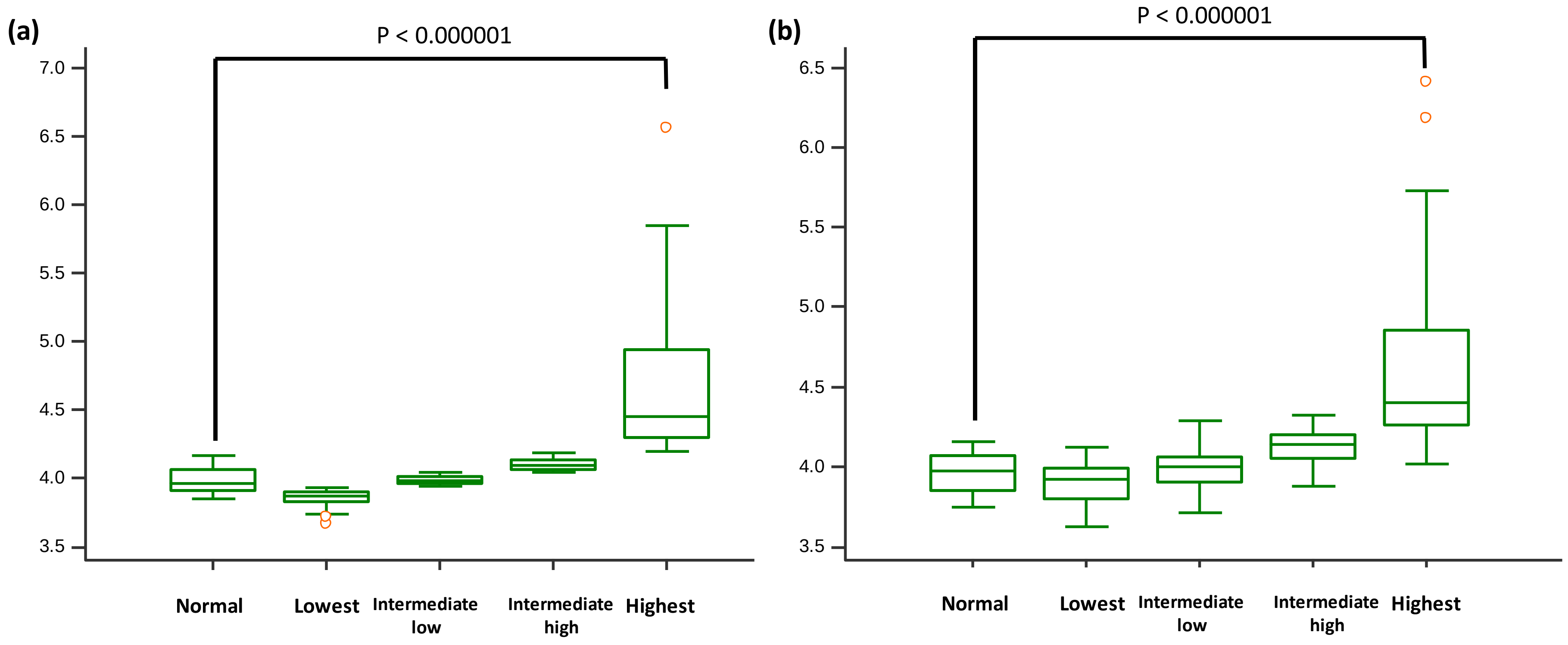

Figure S14. *HOXB-AS3* expressions of MDS patients and health donors. (a) Transcript cluster: TC17002858.hg.1, and **(b)** Transcript cluster: TC17002254.hg.1 on Affymetrix GeneChip® HTA 2.0 arrays.

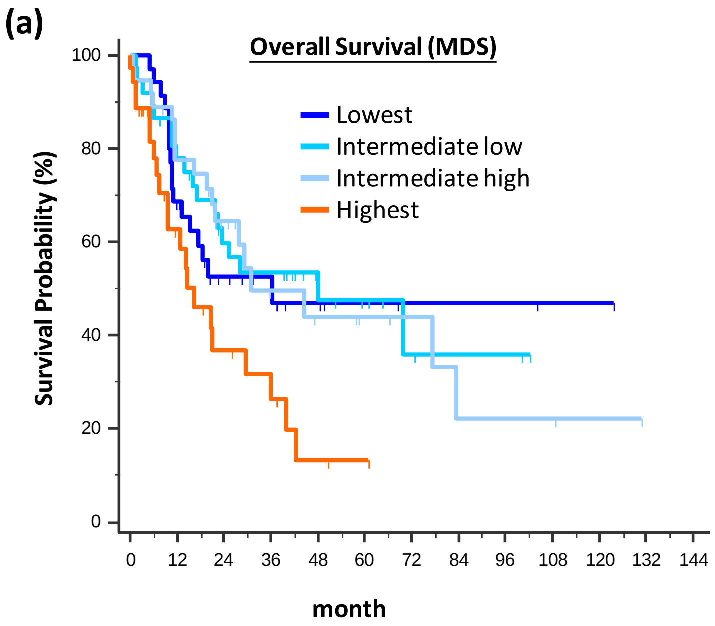

HOXB-AS3 expression	N	Median OS (m)	P value	HR (95% CI)
Lowest	39	36.4	0.0212	1
Intermediate low	40	48.1		0.8633 (0.4671 - 1.5957)
Intermediate high	39	30.9		0.9151 (0.4911 - 1.7055)
Highest	39	16.5		1.9902 (0.9526 - 4.1576)

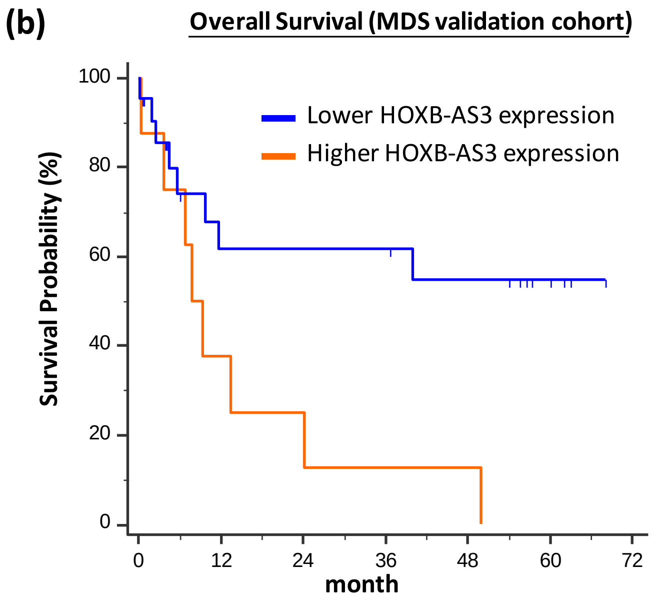

HOXB-AS3 expression	N	Median (m)	P value	HR (95% CI)
Lower	21	NR	0.0147	1
Higher	9	7.8		3.1350 (0.9983 - 9.8449)
Total patients	30	13.5		

Figure S15. Overall survival of MDS patients stratified by the expressions of *HOXB-AS3*. (a) OS in the NTUH MDS training cohort. **(b)** OS in the NTUH MDS validation cohort. The patients were stratified by the expressions of transcript cluster: TC17002254.hg.1 on Affymetrix GeneChip® HTA 2.0 arrays.

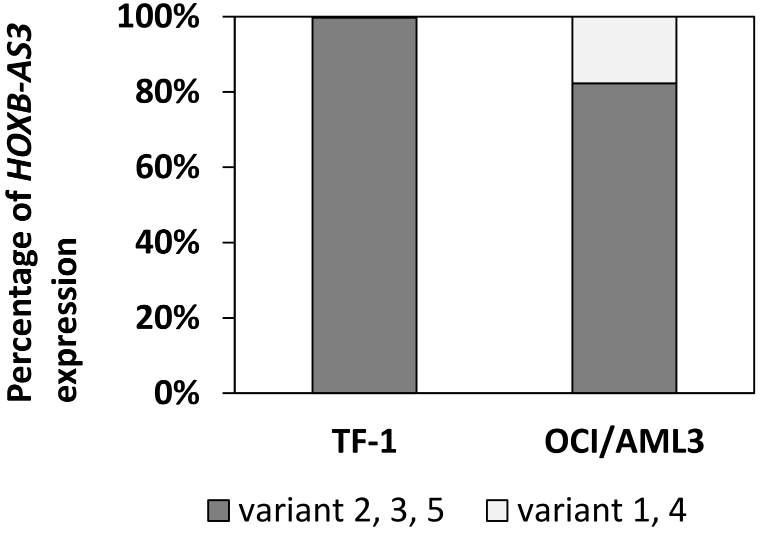

Figure S16. Quantitative PCR analysis of the expressions of different variants in TF-1 and OCI/AML3 cell lines.

Supplementary References

1. Subramanian A, Tamayo P, Mootha VK, Mukherjee S, Ebert BL, Gillette MA, Paulovich A, Pomeroy SL, Golub TR, Lander ES *et al*: **Gene set enrichment analysis: a knowledge-based approach for interpreting genome-wide expression profiles**. *Proc Natl Acad Sci U S A* 2005, **102**(43):15545-15550.

2. Mootha VK, Lindgren CM, Eriksson KF, Subramanian A, Sihag S, Lehar J, Puigserver P, Carlsson E, Ridderstrale M, Laurila E *et al*: **PGC-1alpha-responsive genes involved in oxidative phosphorylation are coordinately downregulated in human diabetes**. *Nat Genet* 2003, **34**(3):267-273.

3. Wang Y, Zhu W, Levy DE: **Nuclear and cytoplasmic mRNA quantification by SYBR green based real-time RT-PCR**. *Methods* 2006, **39**(4):356-362.
